# Phase separation-deficient TDP43 remains functional in splicing

**DOI:** 10.1038/s41467-019-12740-2

**Published:** 2019-10-25

**Authors:** Hermann Broder Schmidt, Ariana Barreau, Rajat Rohatgi

**Affiliations:** 1 Department of Biochemistry, Stanford School of Medicine, Stanford, CA 94305 USA; 2 Department of Medicine, Stanford School of Medicine, Stanford, CA 94305 USA

**Keywords:** Proteins, Cell biology, Molecular biology

## Abstract

Intrinsically disordered regions (IDRs) are often fast-evolving protein domains of low sequence complexity that can drive phase transitions and are commonly found in many proteins associated with neurodegenerative diseases, including the RNA processing factor TDP43. Yet, how phase separation contributes to the physiological functions of TDP43 in cells remains enigmatic. Here, we combine systematic mutagenesis guided by evolutionary sequence analysis with a live-cell reporter assay of TDP43 phase dynamics to identify regularly-spaced hydrophobic motifs separated by flexible, hydrophilic segments in the IDR as a key determinant of TDP43 phase properties. This heuristic framework allows customization of the material properties of TDP43 condensates to determine effects on splicing function. Remarkably, even a mutant that fails to phase-separate at physiological concentrations can still efficiently mediate the splicing of a quantitative, single-cell splicing reporter and endogenous targets. This suggests that the ability of TDP43 to phase-separate is not essential for its splicing function.

## Abstract (web summary)

TDP43 regulates RNA processing and undergoes liquid-liquid phase separation. Here, the authors combine bioinformatic analysis and quantitative measurements of phase properties in living cells to uncover the rules that link the amino acid sequence of TDP43 with its phase properties and find that unexpectedly, mutants of TDP43 that are deficient in phase separation can still function to mediate splicing.

## Introduction

Trans-activating response (TAR) element DNA-binding protein of 43 kDa (TDP43) regulates RNA processing and is found to be aggregated in many neurodegenerative diseases, including amyotrophic lateral sclerosis (ALS), frontotemporal dementia (FTD), and limbic-predominant age-related TDP43 encephalopathy (LATE)^1–4^. One of the best-defined functions of TDP43 is its role in skipping cryptic exons during alternative splicing to ensure that they do not disrupt the final mRNA message^5^. TDP43 predominantly localizes to the nucleus, where it can exist both in a soluble form and in association with various membrane-less protein-RNA condensates thought to form via liquid–liquid phase separation (LLPS)^6–8^.

**Fig. 1:**
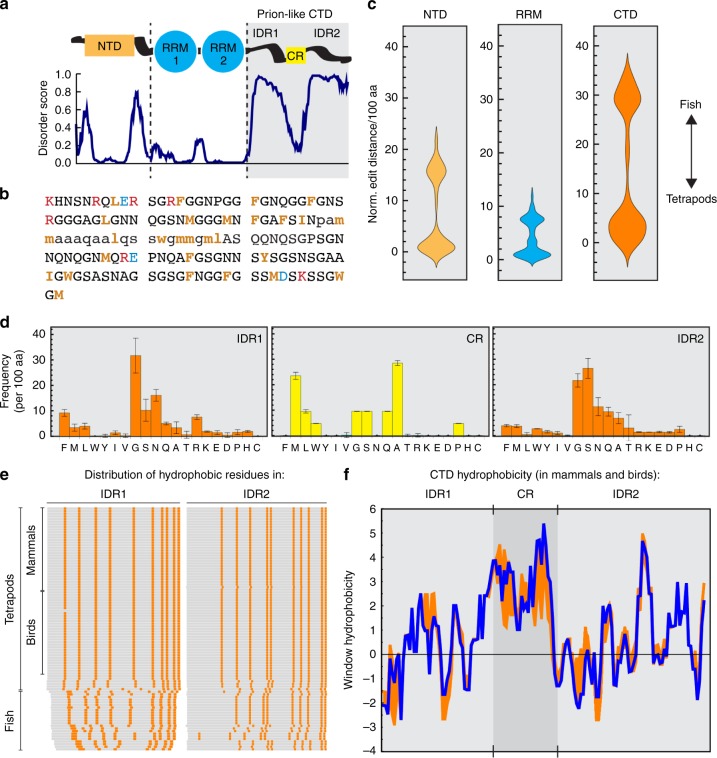
Identification of conserved sequence features in the TDP43 CTD. **a** Disordered regions in TDP43. The RNA-binding protein TDP43 is comprised of a folded N-terminal domain (NTD), two folded RNA-recognition motifs (RRM 1&2) and disorder-containing C-terminal domain (CTD) featuring a highly conserved region (CR). Disorder predictions are based on DisoPred (bioinf.cs.ucl.ac.uk/psipred) analysis of human TDP43 (UniProtKB Q13148). **b** Primary amino-acid sequence of the human TDP43 CTD. Disordered regions are in capital and the CR in lower-case letters. Hydrophobic motifs are highlighted in bold orange, negatively-charged residues in blue and positively-charged residues in red. **c** Violin plots of the sequence divergence (represented as normalized Levenshtein distance per 100 aa) between the NTDs, RRMs, and CTDs of 93 vertebrate TDP43 homologs. The plots show two clusters each, corresponding to fish and tetrapod TDP43 homologs. **d** Amino-acid composition of the intrinsically disordered regions (IDRs) and conserved regions (CRs) that comprise the TDP43 CTD. Plotted are mean amino-acid frequencies per 100 residues ± standard deviation (*n* = 93). **e** Distribution of hydrophobic residues (V, L, I, M, F, Y, W; shown in orange) in the IDRs of 93 vertebrate TDP43 homologs (outlined in gray). Each row represents one homolog. **f** Comparison of the hydrophobicity distribution across mammalian and avian TDP43 CTDs. Hydrophobicity was calculated in a sliding window of five amino acids on the scale of Fauchère and Pliska^24^ (blue trace: human CTD; orange area: deviation amongst CTD homologs)

TDP43 is comprised of a folded N-terminal domain (NTD)^9,10^, two-folded RNA-binding RRM domains^11^ and a low-complexity C-terminal domain (CTD) that is mostly disordered^12–14^. The CTD drives TDP43 phase separation and influences material properties of the resultant condensates^12,14,15^ (Fig. 1a). Remarkably, the CTD harbors nearly all known human mutations in TDP43 that cause ALS^16^. Some of these mutations have been shown to alter the material properties of TDP43 condensates^15,17^ and to interfere with TDP43-mediated splicing^18^, implying that TDP43 phase separation may be required for the function of TDP43 in RNA processing. In contrast, other recent findings indicate that RNA binding prevents TDP43 phase separation^19,20^, suggesting that phase separation may limit or buffer the amount of TDP43 available for its RNA processing functions.

We sought to test these models by exploring the evolutionary sequence space of vertebrate TDP43 CTDs and systematically determining the effect of sequence on both the material properties of TDP43 condensates and on the function of TDP43 in splicing. We find that aromatic residues in the TDP43 CTD are key drivers of phase separation, whereas the material properties of TDP43 condensates depend on aromatic and non-aromatic hydrophobic motifs embedded in a more hydrophilic sequence context. Disease-causing or engineered single amino-acid exchanges that alter this pattern interfere with TDP43 phase dynamics in predictable ways. Notably, even mutations that abrogated TDP43 phase transitions at physiological concentrations failed to prevent the TDP43-dependent splicing of endogenous targets or a quantitative single-cell splicing reporter. These findings suggest that condensate formation is not required for the function of TDP43 in exon skipping.

## Results

### The primary TDP43 CTD sequence is poorly conserved

To assess the role of phase separation in the splicing function of TDP43, we sought to engineer TDP43 phases with custom material properties by mutating the CTD (Fig. 1b) and then measuring the consequences on TDP43-dependent exon skipping. However, in order to customize TDP43 phase properties, we first had to decode the rules that relate TDP43 phase behavior to its amino-acid sequence. Therefore, we turned to evolutionary sequence analysis and compiled a database with protein sequences of 93 vertebrate TDP43 homologs, which we split up into separate collections of NTDs, RRM domains and CTDs. As a simple measure of the sequence diversity within these collections, we calculated the number of amino-acid exchanges, additions or subtractions (i.e., the Levenshtein or edit distance) normalized to a sequence length of 100 amino acids between all members of each category (Fig. 1c). As expected for low-complexity domains, the edit distance in the CTD collection is much higher than in the RRM and NTD collections (mean 21 ± 20 compared to 7 ± 6 and 8 ± 8, respectively), indicating that there is less selection pressure on the precise sequence of amino acids in the CTD. The sequences in each collection fall into either of two clusters (explaining the large mean deviations), which correspond to fish and tetrapod TDP43 homologs. Even though amino-acid substitutions in the CTD have been frequent during vertebrate evolution, point mutations that lead to single amino-acid changes and cause familial ALS in humans are most frequently found in the CTD^21,22^, highlighting the importance of decoding the sequence rules underlying TDP43 phase behavior in physiology and disease.

### Sequence composition and hydrophobic spacing are conserved

Given the poor conservation of the primary amino-acid sequence in the TDP43 CTD, we searched for higher-order sequence patterns that might be conserved. The TDP43 CTD is discontinuous and interrupted by a short, conserved region (CR) prone to adopt an α-helical fold, especially when TDP43 undergoes phase separation^12,15^. This unusual (and complicating) arrangement (Fig. 1a) prompted us to separately analyze the amino-acid composition of the CR and each of the two intrinsically disordered regions (IDRs) that bracket the CR. There was a clear compositional bias in the IDRs and CRs compared to the structured NTD and RRM domains of TDP43, in particular the depletion of charged residues in both types of subdomains (Fig. 1d, Supplementary Fig. 1a). Moreover, the IDRs were especially enriched in glycine residues (~31% in IDR1 and ~21% in IDR2), while alanine (~28%) and methionine (~24%) were abundant and conserved in the CR (Fig. 1d, Supplementary Fig. 1a,b). This agrees with the reported helix propensity of these residues, which is lowest for glycine and high for methionine and alanine^23^.

Beyond overall sequence composition, we noticed that hydrophobic residues (V, L, I, M, F, Y, W) occur in conserved intervals, especially in mammals and birds (Fig. 1e). Indeed, both the number of hydrophobic residues (~10 ± 1 in IDR1 and ~10 ± 1 in IDR2), as well as their relative position and spacing are strikingly conserved in the IDRs (Fig. 1e, Supplementary Fig. 1c). Because the spacer sequences between the hydrophobic motifs are rich in small and polar amino acids (Fig. 1b), the TDP43 IDRs can be characterized as a regular alternating pattern of hydrophobic and hydrophilic segments. To illustrate this periodic hydrophobic pattern, we calculated the hydrophobicity throughout the human TDP43 CTD in a sliding window of five amino acids using fourteen different hydrophobicity scales (Supplementary Fig. 1d). Applying this analysis to our entire set of vertebrate TDP43 CTDs revealed that both the periodicity and the amplitude (i.e., the window hydrophobicity, hereafter shown on the scale of Fauchère and Pliska^24^) of these motifs are well conserved in mammals and birds (Fig. 1f). These findings suggest that the rules governing TDP43 phase separation and dynamics may be encoded in this conserved hydrophobic pattern.

### A strategy to rapidly measure TDP43 LLPS in living cells

**Fig. 2:**
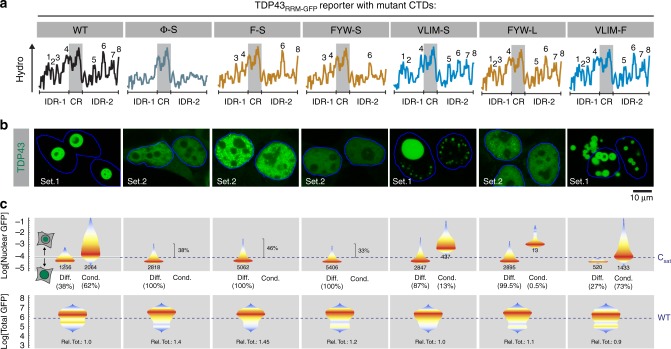
Aromatic residues drive phase separation of TDP43. TDP43_RRM-GFP_ reporter constructs carrying the indicated mutations were transiently transfected into HEK-293T cells and analyzed by high-throughput automated confocal microscopy and flow cytometry. **a** Hydrophobicity plots for each CTD, with numbers highlighting the major hydrophobic clusters. **b** Representative confocal images (63×) of cells transfected with various TDP43_RRM-GFP_ constructs. Blue outline demarcates nuclei; Set1 and Set2 indicate two different photomultiplier gain settings used for optimal display only. **c** Quantification of nuclear TDP43_RRM-GFP_ levels in cells with (‘Cond’) or without (‘Diff’) condensates measured by confocal images (20×) and total TDP43_RRM-GFP_ levels measured by flow cytometry. Data are presented as violin plots, with color-coding representing the probability density of cells in a particular fluorescence bin (blue: lowest probability density; red: highest probability density). Numbers and percentages below each graph reflect the absolute and relative number of transfected cells with (Cond) or without (Diff) TDP43_RRM-GFP_ condensates, respectively. For cells without condensates, percentages within in each graph reflect the number of cells with nuclear TDP43 fluorescence above the estimated critical concentration for phase separation. For total TDP43_RRM-GFP_ levels (bottom row), “Rel. Tot.” denotes the overall median cellular abundance of each CTD variant relative to the WT (*n* ≥ 10,000 cells). Dashed blue lines indicate the C_sat_ of WT TDP43_RRM-GFP_ (C_sat_) or the median total abundance of WT TDP43_RRM-GFP_ (WT), respectively

We sought to measure how changing the periodic hydrophobic motifs in the TDP43 IDRs alters phase behavior in the physiological environment of living cells. However, as we and others have previously observed^15,25^, the small, diffraction-limited nuclear foci formed by endogenous or fluorescently-labeled full-length TDP43 are challenging to analyze by live-cell imaging methods (such as fluorescence recovery after photobleaching or FRAP) required to quantitatively characterize phase properties. To overcome this barrier, we replaced the RNA-binding RRM domains of TDP43 with GFP (Supplementary Fig. 2) to generate a reporter protein (hereafter called TDP43_RRM-GFP_) that assembles into micron-sized liquid droplets (Fig. 2). As we have characterized previously, these TDP43_RRM-GFP_ condensates are amenable to quantitative dynamic analysis in live cells and their phase properties are sensitive to single amino-acid changes in the CTD, including changes that are known to cause ALS^15^. Importantly, the phase behavior of TDP43_RRM-GFP_ droplets resembles that of TDP43 condensates in vitro^12,26,27^ and in neurons^17^. TDP43_RRM-GFP_ condensates are hence ideally suited for rapid, quantitative analysis of CTD sequence variants in live cells, allowing us to screen a panel of CTD mutants with systematic alterations in the composition and spacing of selected hydrophobic motifs or residues.

Notably, we left the CR, previously implicated in stabilizing the phase-separated state^12,28^, untouched in our mutagenesis studies since this region has also been implicated in mediating interactions with other splicing factors such as hnRNPA2^29^. The dual role of the CR makes it challenging to deconvolve effects on protein interactions from effects on phase separation. We used the TDP43_RRM-GFP_ reporter assay to identify mutations in the CTD that lead to alterations in phase separation propensity or phase properties and then introduced these mutations in the context of full-length TDP43 to study their effect on splicing.

### Aromatic residues drive LLPS of TDP43_RRM-GFP_ in cells

Phase separation of TDP43_RRM-GFP_ was completely abrogated (Fig. 2) when we eliminated the hydrophobic motifs in the IDRs by changing all hydrophobic residues (VLIMFYW, hereafter abbreviated as Φ) to serine (all mutant CTD sequences used in this study are listed in Supplementary Table 3). However, the Φ-S mutation also led to the partial mis-localization of TDP43_RRM-GFP_ to the cytoplasm, raising the possibility that the abundance of nuclear TDP43_RRM-GFP_ fell below the critical concentration required for phase separation (C_sat_). To estimate changes in C_sat_ caused by IDR mutations, we combined high-throughput automated confocal microscopy and flow cytometry to monitor the nuclear and total GFP fluorescence in thousands of cells expressing TDP43_RRM-GFP_ with wild-type (WT) or mutant IDRs (Fig. 2). For a population of cells transfected with WT TDP43_RRM-GFP_, comparing nuclear GFP fluorescence in cells with TDP43_RRM-GFP_ droplets to cells with diffuse TDP43_RRM-GFP_ allowed us to estimate the C_sat_ of WT TDP43_RRM-GFP_, which was equal to ≈1/3 of the average expression level in the whole population (which we previously approximated to 5 µM^15^) (Fig. 2). The nuclear abundance of the Φ-S mutant exceeded this threshold concentration in >38% of the cells, yet these cells did not show any signs of droplet formation (Fig. 2). Hence, we conclude that Φ residues are crucial for TDP43 phase separation.

To parse the roles played by the different types of Φ residues, we first mutated all phenylalanine residues, the most dominant hydrophobic side chain in the TDP43 IDRs (Fig. 1d), to serine (F-S). Even though the nuclear abundance of the F-S mutant was above the LLPS threshold in >46% of cells, it failed to form droplets (Fig. 2). Phenylalanine could contribute to phase separation either because of its hydrophobicity, as proposed in the case of nucleoporin FG repeats^30^, or because of its aromatic structure and consequent ability to participate in π-interactions^31^. To assess the relative importance of hydrophobicity or aromaticity, we mutated either all aromatic or all aliphatic residues in the CTD to serine (FYW-S and VLIM-S, respectively). While the FYW-S mutant did not form TDP43_RRM-GFP_ droplets, even in cells where its concentration exceeded the LLPS threshold (Fig. 2), the VLIM-S mutant phase-separated in less than 15% of transfected cells with a significantly elevated C_sat_ (Fig. 2).

Given that there are 11 aromatic residues but only nine aliphatic residues in the TDP43 IDRs, we also converted either all aromatics to leucine (FYW-L) or all aliphatics to phenylalanine (VLIM-F) in order to ensure that changes in phase separation were not merely due to a reduction of overall CTD hydrophobicity. Remarkably, we observed severe phase separation defects in the FYW-L mutant, despite the fact that the overall CTD hydrophobicity was only modestly altered (Fig. 2). In contrast, the VLIM-F mutant formed clumps of small spherical assemblies at nuclear levels considerably below the C_sat_ for WT TDP43_RRM-GFP_ (Fig. 2). Together, our findings suggest that TDP43 phase transitions in cells require aromatic residues. Purely hydrophobic interactions, such as those seen in tropoelastin^32^, are not sufficient.

### Aromatic residues drive LLPS of full-length TDP43 in vitro

**Fig. 3:**
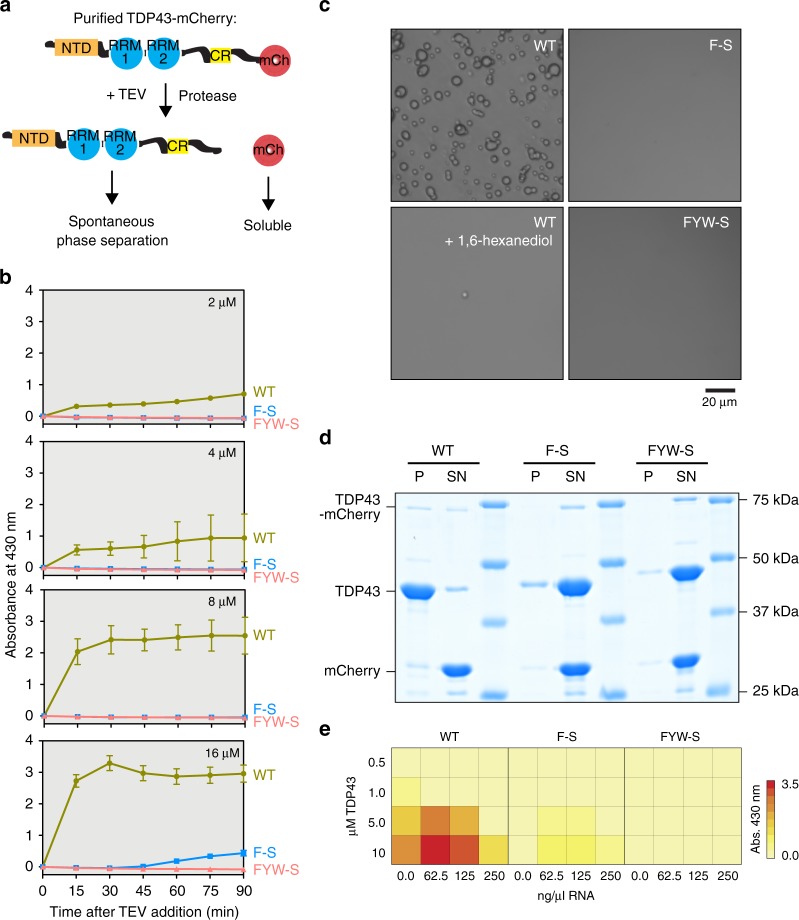
Aromatic residues drive TDP43 phase separation in vitro. **a** Full-length TDP43 variants were initially purified containing C-terminal mCherry tags. To trigger phase separation, tags were cleaved-off by the addition of TEV protease. **b** Phase separation of TDP43 variants at the indicated concentrations was monitored over time after addition of TEV protease by measuring turbidity at 430 nm. Plot markers represent mean values and error bars standard deviation for three replicates per condition. **c** Microscopic analysis confirmed that only wild-type TDP43 phase-separated at 8 µM. Addition of 10% 1,6-hexanediol prevented phase separation. **d** Phase separation of the indicated TDP43 variants at 8 µM analyzed by SDS-PAGE and Coomassie staining after centrifugation at 20,000 × *g* for 30 min. **e** Phase diagram showing TDP43 phase separation in the presence of RNA. Phase separation was monitored by measuring turbidity at 430 nm

To corroborate our findings in the context of full-length TDP43 and further validate our TDP43_RRM-GFP_ reporter assay, we purified full-length TDP43 with WT, F-S or FYW-S variant CTDs from bacteria and investigated their phase separation behavior in vitro (Fig. 3a). As predicted by our reporter assay and previous studies with the isolated CTD^33^, the F-S and FYW-S mutants failed to form condensates even significantly above the physiological TDP43 concentrations of ≈5 µM^19,34^ (Fig. 3b–d). In contrast, WT TDP43 readily phase-separated at 2 µM (Fig. 3b) into condensates sensitive to 1,6-hexanediol (Fig. 3c), an aliphatic alcohol often used to distinguish liquid- and gel-like phases from solid aggregates^35^.

Given that TDP43 is an RNA-binding protein, we next tested the effect of RNA on the phase separation of purified WT and mutant TDP43. As previously reported for other RBPs^27,36^, concentrations of RNA below 250 ng/µl promoted the phase separation of WT and, to a much lesser degree, the F-S mutant TDP43 (Fig. 3e). However, the nuclear RNA concentration in cells is far higher than 250 ng/µl^19^ and at such concentrations, RNA impaired the phase separation of both WT and mutant TDP43 (Fig. 3e), consistent with the model that RNA prevents phase separation of RBPs like TDP43 in the nucleus^19,20^.

In summary, dual analyses of both the phase separation reporter in live cells and purified, full-length TDP43 in vitro showed that aromatic residues drive phase separation of full-length TDP43 in vitro and that the F-S and FYW-S mutants are unable to phase separate at physiological TDP43 and RNA concentrations. Our in vitro data also underscore the usefulness of the TDP43_RRM-GFP_ reporter assay to rapidly screen the effect of CTD mutants on phase separation in cells.

### TDP43_RRM-GFP_ LLPS is largely driven by π–π interactions

**Fig. 4:**
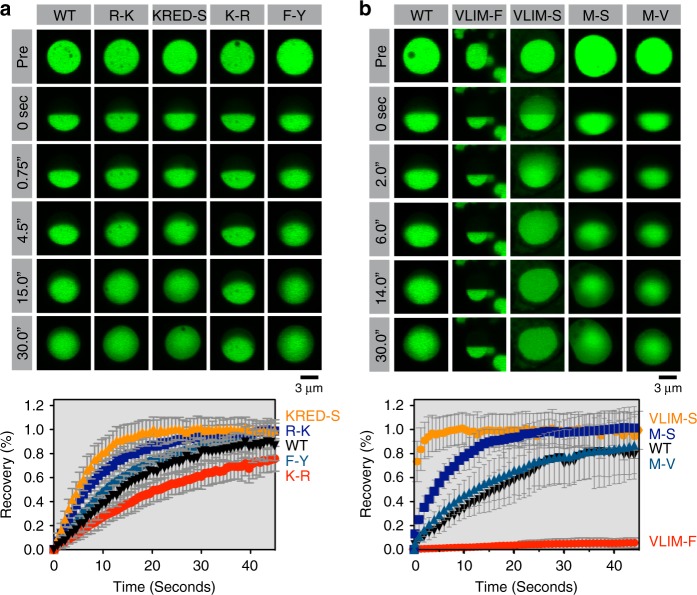
π-interactions are important for phase separation of TDP43. **a**, **b** Half-bleach FRAP experiments to determine the impact of the indicated CTD mutations on the dynamics of TDP43_RRM-GFP_ droplets in transiently transfected HEK-293T cells. Plots show time-dependent, normalized fluorescence recovery. Plot markers represent mean values and error bars standard deviation (*n* ≥ 7)

We next used our live-cell reporter assay to decode the interactions that determine the material properties of TDP43 condensates. In addition to hydrophobic interactions, aromatic residues can also engage in π–π and cation–π interactions. For instance, cation-π interactions, especially involving arginine and tyrosine residues, govern the phase separation of FUS, an RNA-binding protein related to TDP43^31,37–39^. To test the requirement of cation–π interactions for TDP43 phase separation, we mutated all arginine residues to lysine (R-K) or all lysine residues to arginine (K-R). Both mutants formed droplets that were morphologically indistinguishable from WT TDP43_RRM-GFP_ droplets (Fig. 4a). Mutating all charged residues in the TDP43 IDR to serine (KRED-S) failed to abolish phase separation (Fig. 4a). We conclude that neither electrostatic nor arginine-mediated interactions are essential for phase separation of TDP43_RRM-GFP_ in cells.

While certain classes of interactions may have little effect on the formation of droplets *per se*, they could still influence the material properties of these droplets. Material properties of the large TDP43_RRM-GFP_ droplets can be readily assessed using half-bleach FRAP experiments^15^. While the WT and R-K droplets both displayed liquid-like dynamics, the R-K droplets were significantly more dynamic (Fig. 4a), suggesting that arginine-specific interactions (rather than charge-mediated electrostatic interactions) increase the viscosity of TDP43_RRM-GFP_ condensates. Arginine seems to be the most important charged residue for TDP43_RRM-GFP_ droplet dynamics because both the R-K and the KRED-S mutants increased droplet fluidity by a very similar degree (Fig. 4a). Consistent with this view, changing all lysines to arginines (K-R) reduced droplet dynamics (Fig. 4a). Interestingly, mutating all phenylalanine residues to tyrosine (F-Y) did not alter TDP43 phase dynamics (Fig. 4a), suggesting that arginine may engage in similar interactions with both side chains (in contrast to FUS)^37–39^. Together, our data suggest that TDP43 phase transitions are mainly driven by π–π interactions involving aromatic residues, while arginine-mediated interactions influence the dynamics of the resulting condensates.

### Aliphatic residues tune TDP43_RRM-GFP_ droplet properties

The aberrant morphology of VLIM-F condensates (Fig. 2) suggested that an excess of aromatic residues can dramatically alter phase properties. Indeed, the VLIM-F mutant clumps did not behave like liquids. Instead, the complete lack of fluorescence recovery in the bleached half (Fig. 4b) suggested a gel-like or solid-like character. In contrast, the VLIM-S mutant droplets that formed in a minority of cells appeared metastable: they were highly dynamic and almost instantaneously recovered fluorescence in the bleached half (Fig. 4b). Even the selective alteration of methionine residues to serine (M-S) increased TDP43_RRM-GFP_ droplet fluidity, whereas control methionine to valine (M-V) droplets displayed WT-like dynamics (Fig. 4b). Thus, although hydrophobicity is not sufficient to drive TDP43 phase transitions (as shown by the FYW-L mutant, Fig. 2), it can have a dramatic influence on the phase properties of TDP43_RRM-GFP_ condensates. Moreover, our results suggest that a balance between aromatic and hydrophobic residues prevents solidification of TDP43 droplets and ensures that they maintain a liquid-like character. We propose that these requirements drive the conservation of hydrophobic motifs and overall sequence composition in the TDP43 CTD that we observed in our evolutionary sequence analysis (Fig. 1).

### Spacing of hydrophobic residues regulates droplet dynamics

**Fig. 5:**
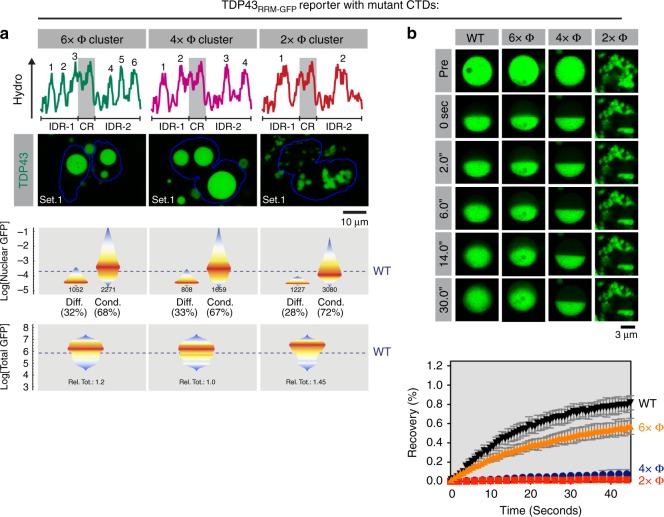
The spacing of hydrophobic CTD residues dictates TDP43_RRM-GFP_ condensate properties. **a** Hydropathy plots (with numbers denoting hydrophobic peaks), cellular distribution, nuclear abundance and total cellular abundance of TDP43_RRM-GFP_ variants with altered spacing between hydrophobic peaks. Data are represented as in Fig. 2 (*n* ≥ 2400 and 10,000 cells for nuclear and total GFP levels, respectively), except that dashed blue lines represent the median nuclear GFP levels of cells with WT TDP43_RRM-GFP_ condensates or the median total abundance of WT TDP_RRM-GFP_ (see Fig. 2). See Supplementary Fig. 2 for an illustration of how the mutants were constructed by sliding hydrophobic clusters. **b** Material properties of condensates formed by TDP43_RRM-GFP_ variants probed by half-bleach FRAP (as in Fig. 2b, c). Plot markers represent mean values and error bars standard deviation (*n* ≥ 5)

An unexpected finding of our evolutionary analysis was the identification of conserved spacing between the hydrophobic motifs in the TDP43 CTD (Fig. 1e). In the CTD of human TDP43, both IDRs contain four major hydrophobic clusters each (Fig. 2). These clusters are reminiscent of ‘stickers’ in a model of associative polymers suggesting that both the number and the individual adhesive strength of stickers determine polymer phase separation^40^. To test the relative importance of these two parameters, we designed mutants where the total number of clusters was reduced from eight to six, four or two by simply grouping the clusters together (Fig. 5a, Supplementary Fig. 3a). Thus, we reduced the number of stickers while simultaneously increasing their hydrophobicity, without changing the amino-acid composition or overall hydrophobicity of the CTD.

Whereas changing the number of hydrophobic (Φ) clusters from eight (WT) to six (6 × Φ) or to four (4 × Φ) did not change the morphology of TDP43_RRM-GFP_ reporter droplets, TDP43 variants with only two Φ clusters (2 × Φ) formed amorphous assemblies that frequently mis-localized to the cytoplasm and behaved like solids in half-bleach experiments (Fig. 5a). Live-cell imaging suggested that the 2 × Φ mutant immediately aggregates after translation, as we observed a much earlier onset of phase transition (without a clearly detectable initial soluble state) and a greatly increased number of initial foci compared to WT (Supplementary Fig. 3b). While the 6 × Φ and 4 × Φ mutants formed spherical droplets suggestive of a liquid-like genesis, the 6 × Φ mutant had reduced dynamics and the 4 × Φ droplets behaved like solids lacking any intra-droplet redistribution of TDP43_RRM-GFP_ (Fig. 5b). Interestingly, the solid 4 × Φ mutant also partially mis-localized to the cytoplasm. Although we decreased the number of hydrophobic patches in the 6 × Φ, 4 × Φ, and 2 × Φ mutants, increasing the adhesiveness of each patch made the mutant phases more solid.

Taken together, our mutagenesis studies support a role for both the number and the spacing between aromatic and aliphatic residues across the TDP43 IDRs in tuning intra-phase dynamic and preventing droplet solidification, aggregation and cytoplasmic mis-localization. We speculate that evolutionary conservation of both the number and spacing of these hydrophobic clusters (Fig. 1e, Supplementary Fig. 1c) reflects the need to tune condensate dynamics within an optimal range while minimizing the risk of aggregate formation and mis-localization.

### The effect of point mutations can be predicted and rescued

**Fig. 6:**
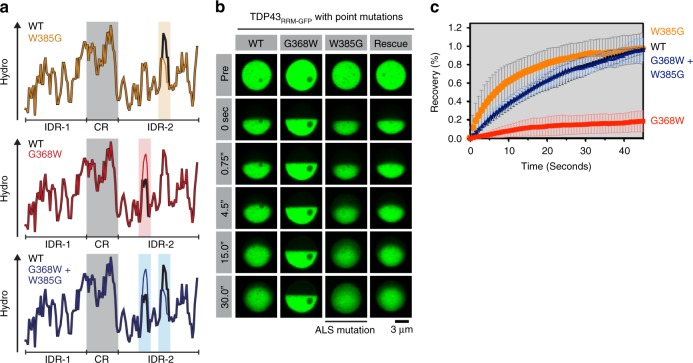
Compensatory mutations rescue altered phase dynamics caused by primary mutations. **a** Plots showing the effects of single- and double-point mutations on local hydrophobicity in the TDP43 CTD. **b**, **c** Material properties of TDP43_RRM-GFP_ droplets with single and double CTD mutations formed by transient transfection in HEK-293T cells. Plot markers represent mean values and error bars standard deviation (*n* ≥ 10)

To further test our periodic hydrophobicity model, we sought to determine if it could predict the effect of ALS-associated and engineered IDR mutations on the phase properties of TDP43_RRM-GFP_ droplets. We first focused on tryptophan 385, which is mutated to glycine in ALS patients^41^ and forms the core of the major hydrophobic peak in IDR2 (Fig. 6a). The effects of the W385G mutation on TDP43 phase properties are not known. We expected that changing tryptophan 385 to glycine would enhance the internal dynamics of TDP43 condensates because it significantly reduces the hydrophobicity of IDR2 (Fig. 6a). This prediction conflicts with the prevalent view that disease mutations in RBPs promote the conversion of liquid phases into more solid-like assemblies^27,36,42,43^. In accordance with our model, we observed faster fluorescence recovery kinetics in half-bleached W385G mutant TDP43 condensates (Fig. 6b, c). Thus, disease mutations in IDRs can also increase the liquidity of condensates, not just enhance their solid-like nature as commonly proposed.

Next, we studied another ALS-associated mutation, G348V^16^. This mutation increases the amplitude of a hydrophobic peak in IDR2 and, as expected, makes TDP43 phases less dynamic in half-bleach experiments (Supplementary Fig. 4a, b). The effect of the G348V mutation was comparable to that of a synthetic G348F mutation (Supplementary Fig. 4a, b), supporting the notion that hydrophobicity changes alone (without changes in aromaticity) can influence TDP43 phase properties. Similarly, increasing the height of a peak centered around glycine 309 by mutating it to phenylalanine, the most common hydrophobic residue found in the TDP43 IDR (Fig. 1d), decreased phase dynamics in agreement with our model (Supplementary Fig. 4c, d). A control mutation of glycine 309 to serine, which did not change local hydrophobicity, had no effect on TDP43 phase dynamics (Supplementary Fig. 4c, d).

As the most stringent test of our model that periodic hydrophobic motifs drive phase behavior, we aimed to rationally make second-site suppressor mutations that would rescue or restore the altered phase dynamics produced by a primary mutation. Guided by our model, we sought to reverse the fluidizing effect of the W385G mutation on TDP43_RRM-GFP_ condensates by increasing hydrophobicity at a distant site. We chose the minor hydrophobic peak around glycine 368, because mutating this residue to tryptophan would restore both the overall hydrophobicity and the amino-acid composition of the CTD (Fig. 6a). As expected, the elevated hydrophobicity produced by the G368W mutation in isolation makes TDP43 condensates markedly more solid (Fig. 6b, c). However, when the G368W mutation is made in combination with the W385G mutation, wild-type phase dynamics are restored in the double mutant (Fig.6b, c). Taken together, our findings demonstrate that the intra-phase dynamics of TDP43 droplets can be tuned in predictable ways by single amino-acid exchanges and suggest that the consequences of these mutations can be predicted based on how they influence local hydrophobicity.

### A quantitative reporter assay to measure exon skipping

Having decoded the rules that govern TDP43 condensate formation and dynamics in cells put us in a position to systematically test the model that TDP43 function in RNA processing requires phase separation. We decided to focus on the role of TDP43 in exon skipping during alternative splicing, because it (i) has been intensively studied^5,44,45^, (ii) is purportedly dependent on the CTD^29,46,47^, and (iii) is disrupted by ALS-associated mutations in the CTD^18^. In addition, it was recently demonstrated that splicing regulation by the low-complexity domain-containing alternative splicing factor Rbfox depends on its phase separation^48^.

**Fig. 7:**
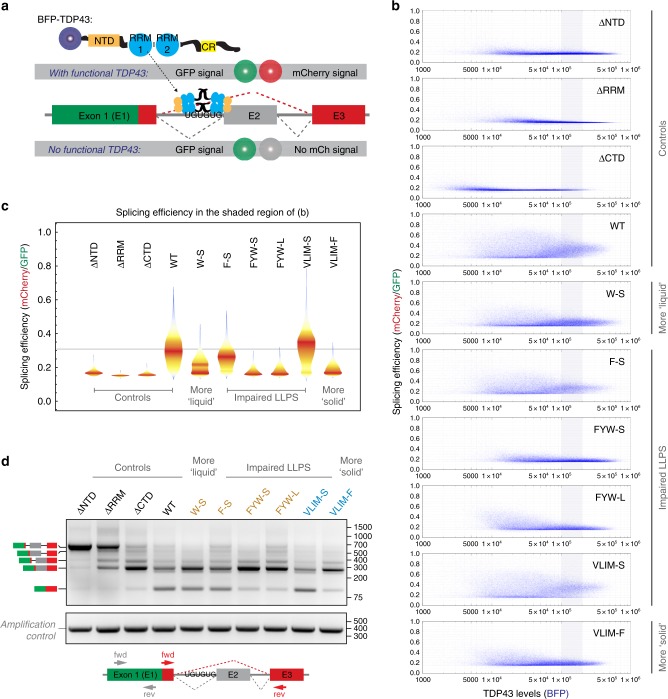
LLPS-deficient TDP43 supports exon skipping in a single-cell splicing reporter. **a** Splicing reporter design. Production of a dually-fluorescent GFP-mCherry fusion protein depends on the presence of functional full-length TDP43 to mediate skipping of exon 2 (“E2”, derived from the TDP43-regulated exon 9 of the CFTR gene). TDP43 and its CTD variants contain N-terminal BFP tags. BFP-TDP43 and the GFP-mCherry splicing reporter are expressed from the same plasmid via a bidirectional promoter by transient transfection in TDP43−/− HEK-293T cells. **b** Splicing efficiency of the indicated TDP43 compositional mutants quantitatively measured on a single-cell level by flow cytometry. Splicing efficiency is expressed as the mCherry/GFP ratio per cell and plotted against TDP43 abundance, measured by BFP fluorescence. **c** Splicing efficiency of the indicated TDP43 mutants in cells selected to have similar expression levels (derived from the region highlighted in **b** by blue shading). Data are presented as violin plots, with color-coding representing the probability density of cells with a particular mCherry/GFP ratio. The gray horizontal line indicates the median mCherry/GFP ratio for WT TDP43. **d** Exon-skipping activity of TDP43 variants measured by RT-PCR using primers spanning exon 2 (see cartoon below the gels). Diagrams to the left denote the electrophoretic positions of the various splicing intermediates and products. GAPDH was used as amplification control

To analyze the splicing activity of TDP43 variants rapidly, quantitatively and with single-cell resolution, we built a flow cytometry-compatible splicing reporter, which uses a bidirectional promoter to co-express a splicing-sensitive fluorescent protein and full-length TDP43 (or variants thereof) from the same transiently transfected plasmid (Fig. 7a). The splicing module is a full-length mEGFP fused to an mCherry construct interrupted by exon 9 of the CFTR gene^49^, which is skipped only in the presence of functional TDP43 (Fig. 7a, Supplementary Fig. 6a). We established that the splicing of this module to express a functional mCherry was dependent on TDP43 by transfecting it into WT and TDP43−/− HEK-293T cells, which we generated by introducing loss-of-function mutations in the *TARDBP* gene using Crispr/Cas9-mediated editing (Supplementary Fig. 5). Skipping of exon 9 was severely reduced in TDP43−/− cells, assayed using either flow cytometry or reverse transcription-PCR (RT-PCR) to directly measure the distribution of splicing intermediates (Supplementary Fig. 6). For our assays, this splicing module was co-expressed in TDP43−/− cells with full-length TDP43 CTD mutants fused to an N-terminal BFP tag, allowing for quantification of cellular TDP43 levels. To rigorously control our assay, the efficiency of TDP43-mediated exon skipping (mCherry fluorescence) was normalized to the expression level of the splicing reporter (GFP fluorescence) and plotted against the total expression levels of the re-introduced TDP43 (BFP fluorescence) on a cell-by-cell basis (Fig. 7b).

### IDR-driven condensation is dispensable for exon skipping

We first used our splicing reporter assay to compare the splicing efficiency of exogenous full-length TDP43 (WT) to TDP43 variants lacking the RNA-binding domains (∆RRM), the N-terminal domain (∆NTD) or the C-terminal domain (∆CTD). Re-expression of TDP43_WT_ in TDP43−/− cells rescued the exon-skipping defect, whilst TDP43_∆RRM_ was non-functional (Fig. 7b–d). Both TDP43_∆NTD_ and TDP43_∆CTD_ also showed splicing defects (Fig. 7b–d), as previously reported^29,46,50^. Notably, splicing efficiency (mCherry/GFP ratio) became less variable across the population of individual cells and converged towards a set-point as the TDP43_WT_ protein levels (BFP fluorescence) increased (Fig. 7b).

We next studied the F-S CTD mutant, which failed to phase separate at physiological concentrations in vitro (Fig. 3b) or when introduced into the TDP43_RRM-GFP_ reporter (Fig. 2). Nevertheless, this mutant could still support splicing, regardless of whether exon skipping was assessed by flow cytometry (Fig. 7b, c) or RT-PCR (Fig. 7d). However, we did observe a small reduction in splicing efficiency (Fig. 7b, c): while the amount of correctly spliced, exon-excluded product was equal in cells transfected with WT or F-S TDP43, the F-S cells expressed a higher amount of the product in which the exon was retained (Fig. 7d). Similar to WT TDP43, splicing efficiency also became less variable across a population of cells as the protein levels of the F-S mutant increased (Fig. 7b). These unexpected observations suggest that phase separation is not necessary for the function of TDP43 in exon skipping, but may enhance splicing efficiency.

To understand how material properties of condensates may influence splicing, we tested the metastable VLIM-S (Figs. 2, 4) and the phase separation-deficient FYW-S and FYW-L mutants (Figs. 2, 3). Whereas the VLIM-S mutant slightly outperformed WT TDP43 in our splicing assay, the FYW-S and FYW-L mutants showed a splicing defect that was almost as severe as the complete loss of the CTD (Fig. 7b–d). To distinguish whether this is due to a loss of phase separation propensity or simply because aromatic residues are required for splicing, we mutated the only two tryptophan residues in IDR2 (Fig. 1b) to serine. This W-S mutation did not abolish phase separation, but rendered TDP43_RRM-GFP_ droplets more liquid (Supplementary Fig. 7). Strikingly, the W-S mutant caused a much more pronounced splicing defect than the F-S mutant (Fig. 7b–d), which is entirely deficient in phase separation (Figs. 2, 3). These findings suggest that aromatic residues, especially tryptophan, may contribute to TDP43 splicing function independently of their role in phase separation. Yet, increasing the number of aromatics in the VLIM-F mutant caused splicing defects (Fig. 7b–d), perhaps because it leads to sequestration of TDP43 into solid aggregates in the cytoplasm (Figs. 2,4).

In summary, our data show that the effects of mutations in the IDR on TDP43 phase behavior and splicing function are not correlated, thus supporting the conclusion that IDR-driven phase separation is not essential for the function of TDP43 in exon skipping.

### Impaired phase separation does not block endogenous splicing

Given that our results disagreed with the prevalent view that phase separation plays a functional role in splicing, we also tested the ability of various TDP43 mutants to support exon skipping in endogenous mRNA targets. Therefore, we re-introduced GFP-tagged, full-length TDP43 variants into TDP43−/− HEK-293T cells by stable transduction. We estimated the nuclear TDP43 concentrations in our add-back lines to be 2–4 µM (Supplementary Fig. 8), which is in the expected physiological range^19,34^ where WT, but not the F-S and FYW-S mutants, could phase separate (Fig. 3).

**Fig. 8:**
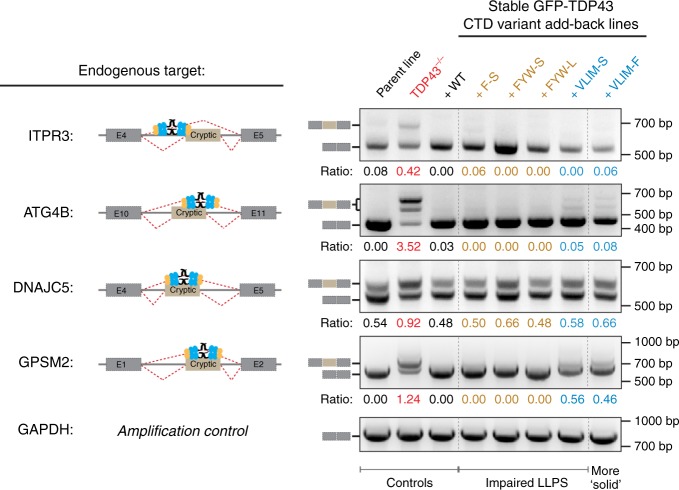
LLPS-deficient TDP43 supports exon skipping in endogenous genes. Splicing of cryptic exons in *ATG4B, DNAJC5, GPSM2*, and *ITPR3* known to be regulated by TDP43 was assessed in WT, TDP43−/−, or TDP43−/− HEK-293T cells stably expressing the indicated variants of GFP-TDP43. See Supplementary Fig. 8 for characterization of these cell lines, including estimation of nuclear GFP-TDP43 levels. Cartoons to the left show the position of the TDP43 binding site relative to the cryptic exon in each gene. Primers aligning to exons flanking the cryptic exon were used in RT-PCR (gel on right) to assess the relative abundance of the mRNA species that either retained or excluded the cryptic exon. The expected PCR products for the correctly spliced transcripts are marked to the left of the gels. Numbers below each lane indicate the ratio of the exon-included to exon-excluded (i.e., correctly spliced) product GAPDH was used as amplification control

We selected four previously validated endogenous targets (*ATG4B*, *DNAJC5, GPSM2*, and *ITPR3*) that contain cryptic exons (CEs) suppressed by TDP43^5,44^ for our analysis. In contrast to our splicing reporter assay, in which both TDP43 and the splicing target are overexpressed proportionally, we analyzed the endogenous mRNAs expressed from these genes by RT-PCR for the presence of the relevant cryptic exons (Fig. 8). As expected, we observed an increase of transcripts containing CEs at the expense of the fully spliced product in TDP43−/− cells (Fig. 8). Re-introduction of WT TDP43 restored the skipping of CEs in the mRNAs of all four targets (Fig. 8). Consistent with our results using the splicing reporter assay (Fig. 7), the F-S mutant supported the skipping of CE to a similar degree as WT TDP43 (Fig. 8). Notably, the FYW-S and FYW-L mutants (which cannot phase separate, Fig. 2) also showed significant splicing activity in this assay (Fig. 8). Indeed, we only observed a splicing defect in the *GPSM2* RNA for the VLIM-S and VLIM-F mutants (Fig. 8). Taken together, these findings further support the notion that condensate formation is not required for TDP43-mediated exon skipping.

## Discussion

Phase transitions of proteins and nucleic acids into biomolecular condensates in cells are emerging as an important cellular mechanism to dynamically organize biochemical reactions in space and time^51–53^. For example, proteins with the ability to phase separate are involved in all steps of gene expression^54^, where LLPS has been suggested to play pivotal roles in chromatin organization^55,56^, transcription^57^ and alternative splicing^48^. Many proteins that display phase separation behavior contain low-complexity regions such as the TDP43 CTD. While phase separation has been observed and phase behavior characterized for many proteins both in vitro and in vivo, the molecular code that relates amino-acid sequence to phase behavior often remains enigmatic^58^. This has been a major barrier to understanding the functions of TDP43, an abundant RNA-binding protein that is mutated and aggregated in neurodegenerative diseases.

Using a systematic mutagenesis approach rooted in evolutionary sequence analysis, we uncovered that aromatic residues drive the phase separation of TDP43 (Figs. 2, 3), whereas a conserved sequence pattern dictates the material properties of the resultant TDP43 condensates (Figs. 4, 5, 6). TDP43 contains two IDRs in its CTD, which both feature aromatic and aliphatic residues with a conserved spacing that are embedded in a flexible, polar sequence context (Fig. 1). The spacing of these ‘cohesive’ residues seems to be critical for maintaining TDP43 phase properties and providing a barrier to solidification (and ultimately aggregation), as mutations that reduce the spacing, while conserving overall IDR hydrophobicity, make TDP43 condensates more solid-like (Fig. 5).

In the case of IDR-mediated phase separation, significant uncertainty (and controversy) surrounds the identity of the structural motifs that mediate favorable cohesive interactions within condensates and hence determine their dynamics (or their liquid-like or solid-like character). Two models for such structural motifs include (1) multivalent interactions mediated by aromatic, charged or polar residues^31,59–61^, and (2) loosely adherent cross-β structures that lack extended hydrophobic segments and hence fail to form insoluble amyloid aggregates^62–65^. Examples of the latter are low-complexity, aromatic-rich, kinked segments (or LARKs) that have been proposed to form distorted β-sheets stabilized by inter-strand hydrogen bonds or aromatic stacking interactions^62,63,66^.

Although the TDP43 IDRs contain aromatic residues (especially phenylalanine), only a few hydrophobic motifs in IDR1 are predicted to be LARK segments^62,66^ (Supplementary Fig. 9). This is in contrast to other RNA-binding proteins that form biomolecular condensates, such as FUS or hnRNPA1^27,36,42,43^, which are among the cellular proteins with the highest predicted LARK content^62,63^. It is also clear that mutations in IDR2, which is not predicted to contain any LARKs, can have dramatic effects on TDP43 phase dynamics (Fig. 6). While our sequence analysis and mutagenesis studies do not offer atomic insights to distinguish between these two structural models, our work demonstrates that regularly spaced π–π, hydrophobic and arginine-mediated interactions are determinants of TDP43 phase separation and behavior in cells. Thus, it appears that the CTD features multiple strategies to restrain TDP43 aggregation potential while maintaining the ability to phase separate, including optimized spacing of cohesive motifs, limiting LARK motifs and, as previously demonstrated, containing a conserved helical region (CR) that interrupts its IDR^12^. Interestingly, while the CR has been shown to contribute to TDP43 condensate formation^12,15,28^, our in vitro data shows that it is not sufficient to drive the phase separation of full-length TDP43 lacking cohesive IDR residues at physiological concentrations (Fig. 3).

Based on our mutagenesis studies, we developed a heuristic model to guide the design of mutations that change the dynamics of TDP43 phases in predictable ways (Fig. 6). Such systematic manipulation of the molecular code underlying TDP43 phase behavior should facilitate future investigations on how phase dynamics influence the various functions of TDP43 in physiological models. Surprisingly, we find that CTD-driven phase separation is dispensable for the function of TDP43 in exon skipping (Figs. 7, 8). We identified the F-S mutant as the minimal variant required to abolish phase separation of both our TDP43_RRM-GFP_ reporter (Fig. 2) as well as of purified, full-length TDP43 at physiological concentrations (Fig. 3) without disrupting the CR that mediates important interactions with other splicing factors^29^. Despite this profound defect, the F-S mutant could still support splicing of all tested endogenous targets at a level similar to WT TDP43 (Fig. 8) and was almost as effective as WT TDP43 in supporting splicing of our synthetic reporter (Fig. 7). We would have predicted a defect comparable to that seen in the ∆RRM or ∆CTD deletion mutants if phase separation were indeed required for splicing function (Fig. 7). However, we note that the F-S mutant is slightly less active than WT TDP43 in supporting splicing of our synthetic reporter and the VLIM-S and VLIM-F mutants show partial defects in supporting splicing of one of the endogenous mRNA targets (*GPSM2*, Fig. 8). Hence, we cannot exclude the possibility that the splicing efficiency of some targets may be influenced by the phase properties of TDP43.

The view that phase separation is not required for splicing function is consistent with prior work showing that the exon-skipping function of TDP43 is maintained when the CTD is replaced by a heterologous splicing-suppressor domain^5^ and further supported by the recent observations that RNA-binding proteins are often prevented from phase separating in the nucleus by high concentrations of RNA^19,20^. Thus, strategies to solubilize TDP43, such as antisense oligonucleotides^67^ or small molecule inhibitors^26,68^ that prevent the accumulation and aggregation of TDP43, may also restore splicing defects in ALS patients. Despite a decade of work on this important protein involved in ALS, the central question of how phase separation and phase dynamics regulate the physiological function of TDP43 remains a challenge for the future. Further studies will be required to tested whether phase separation regulates other functions of TDP43 function in motor neurons, including axonal mRNA transport^17,69^.

## Methods

### Cell culture

HEK-293T were obtained from ATCC and cultured at 37 °C and 5% CO_2_ in high-glucose DMEM (GE Healthcare) supplemented with 10% FBS (Atlanta Biologicals), 2 mM L-glutamine (Gemini Biosciences), 1x MEM non-essential amino acids solution (Gibco), 40 U/ml penicillin and 40 µg/ml streptomycin (Gemini Biosciences).

To generate HEK-293T TDP43 knock-out cells, sgRNAs (see Supplementary Table 1) targeting TDP43 were cloned into pX458 (Addgene #48138), transfected into HEK-293T cells using X-tremeGENE9 (Roche) and single-cell sorted for GFP-positive cells using a Sony SH800 flow cytometer. Individual clones were screened by immunoblotting using α-TDP43 (Proteintech #10782-2-AP; 1:5,000 dilution) and α-alpha-tubulin (Sigma T6199; 1:10,000 dilution) primary antibodies with IR680LT donkey α-mouse (LiCor #926–68022; 1:10,000) and IR800CM donkey α-rabbit (LiCor #926–32213) secondary antibodies for detection with a LiCor Odyssey imager. Single-cell clones lacking TDP43 protein were further analyzed by qPCR after total RNA extraction with Trizol (Invitrogen), cDNA synthesis with the iScript kit (Bio-Rad) and quantification with the SYBR green kit (Bio-Rad) on a QuantStudio 5 real-time PCR system (Thermo Fisher Scientific). qPCR primers are listed in Supplementary Table 1. HEK-293T TDP43 knock-out cells were cultured as the parent HEK-293T cells.

To generate stable HEK-293T cell lines, the indicated GFP-TDP43 CTD variants were cloned into pLenti-CMV Puro DEST (Addgene #17452) and transfected into HEK-293T cells together with pMD2.G (Addgene #12259) and psPAX2 (Addgene #12260) to produce lentiviral particles for transduction. Virus was harvested 48 and 72 h after transfection, pooled, filtered through non-binding 45 µm syringe filters (Pall Corporation) and used to transduce HEK-293T TDP43 knock-out cells. After 24 h, the virus-containing medium was removed and replaced with selection medium containing 2 µg/ml Puromycin (Sigma–Aldrich). After 7 days of selection, cells were bulk sorted for similar GFP levels (where possible) using a Sony SH800 flow cytometer. See Supplementary Fig. 8 for comparison of nuclear GFP-TDP43 levels in stable add-back lines after sorting and Supplementary Fig. 10 for sort gates. All stable HEK-293T GFP-TDP43 cell lines were cultured as the parent HEK-293T cells.

### Bioinformatics analysis

Source code is available on Github (https://github.com/RohatgiLab/TDP43-analysis). In short, the NCBI blastp suite was used to retrieve 394 vertebrate TDP43 homologs, with human TDP43 (UniProtKB Q13148 [https://www.uniprot.org/uniprot/Q13148]) as the query in a search limited to taxid:7742. A species and isoform filter was applied to remove sequence redundancy, reducing the dataset to 101 TDP43 homologs. Based on sequence alignments to the well-characterized RNA-binding domain of human TDP43 (PDB:4bs2^11^), the dataset was split up into sub-collections of N-terminal domains, RRM domains and C-terminal domains. The latter was further dissected into the IDR and CR collections using sequence alignments to the previously described CR^12,15^. To determine the density distribution of hydrophobic residues in the IDRs (but not CRs), we calculated the number of V, L, I, M, F, Y, and W residues in each IDR and normalized the values to a reference length of 100 amino acids. Hydrophobicity was calculated according to the scale of Fauchère and Pliska^24^, which we re-scaled to range between 0 and 1 for clarity. For the comparison of hydrophobicity scales in Supplementary Fig. 1c, the ProtScale tool from the ExPASy suite was used.

### DNA constructs

All plasmids used in this study are summarized in Supplementary Table 2. Sequences and maps are made available on Addgene. All compositional IDR mutants were designed in silico, synthesized as gBlocks by Integrated DNA Technologies and introduced into TDP43 using Gibson cloning. IDR point mutations were introduced by Gibson-based site-directed mutagenesis.

### Phase separation reporter assay

HEK-293T cells were seeded onto acid-washed coverslips coated with 0.1% gelatin (Sigma–Aldrich) in 24-well plates at 5 × 10^4^ cells/well, allowed to adhere overnight and transfected with the indicated reporter constructs (500 ng/well) using X-tremeGENE 9 (Roche). Twenty four hour after transfection, cells were fixed with 4% PFA for 15 min at room temperature, washed 3× with PBS and permeabilized with 0.1% Triton X-100 (Sigma–Aldrich) in PBS for 15 min at room temperature. Cells were then washed 3× with PBS and mounted onto glass slides using ProLong Diamond mounting medium containing DAPI (Molecular Probes). After curing overnight at room temperature, z-stacks were automatically taken for all samples at 16 randomly-selected positions across each coverslip with a Leica DMI-6000B microscope equipped with a Yokogawa CSU-W1 spinning disk confocal unit at ×20 resolution. A 488-nm laser was used for GFP excitation and an Andor iXon Ultra DU888 EMCCD camera for signal detection.

For quantification, custom-written Mathematica pipelines were used that are available on Github (https://github.com/RohatgiLab/TDP43-analysis). In short, the ImageFocus-Combine function of Mathematica (Wolfram Research) was first used to make z-projections for each acquired z-stack. Using the DAPI channel, nuclear masks were then generated and used to (i) extract single nuclei and (ii) measure nuclear GFP levels. To subsequently compare the GFP levels of TDP43 droplet-free or droplet-containing nuclei, the morphological component analysis package of Mathematica was used to automatically detect spherical objects and classify the extracted nuclei accordingly. Mis-classifications where manually corrected if necessary. Non-transfected cells (within the population of transfected cells) were excluded from analysis by filtering based on the nuclear GFP signal of a non-transfected control cell population. At least 2000 nuclei per sample were quantified.

To compare total reporter expression levels, HEK-293T cells were seeded in 24-well plates at 5 × 10^4^ cells/well, transfected as above, harvested 24 h later and at least 10,000 analyzed by flow cytometry with a BD Accuri C6 instrument (excitation laser: 488 nm, emission filter: 586/40 nm). A graphical overview of the gating strategy can be found in Supplementary Fig. 11.

### Phase dynamics reporter assay

HEK-293T cells were seeded into eight-well ibidi µ-slides (3 × 10^4^ cells/well), allowed to adhere overnight and transfected with the indicated reporter constructs (300 ng/well) using X-tremeGENE 9 (Roche). Twenty four hour later, the culture medium was exchange with L-15 medium (Gibco) supplemented with 10% FBS, and cells imaged using a Leica SP8 laser-scanning confocal microscope equipped with a 488-nm laser, ×63 glycerol objective (NA 1.3) and a temperature-controlled incubation chamber (Life Imaging Services) set to 37 °C.

Half-bleach experiments were recorded and quantified using the FRAP module of the Leica Application Suite X software. To ensure that only similarly-sized droplets of comparable intensities were bleached, the same bleach window and detector settings were used. For each recorded time point (t), the fluorescence intensities within the bleached droplet hemisphere were then normalized to the fluorescence intensity of the corresponding unbleached droplet hemisphere. These normalized, time-dependent fluorescence intensities I_t_ were then used to calculate the fluorescence recovery (FR) according to the following formula:

1 $${\mathrm{FR}}\left( {\mathrm{t}} \right) = \left( {{\mathrm{I}_t}} - {I_{\mathrm{t0}}} \right)/\left( {\mathrm{I}}_{\hbox{before bleaching}} - {\mathrm{I}_{t}} - {I_{\mathrm{t0}}} \right)$$

with t0 being the first time point observed after photobleaching. At least five individual droplets from different cells were analyzed and GraphPad Prism was used to plot replicate measurements (mean ± SD). WT reporter constructs were included in all experiments as a control for comparison across dataset. For all measurements and initial fluorescence signal quantifications, investigators were blinded to prevent bias.

### Live-cell imaging of TDP43 phase formation

To follow TDP43 reporter phase formation over time, cells were transfected as above and imaged in L15 medium (Gibco) supplemented with 10% FBS at 37 °C every hour for a total of 24 h after transfection with a Leica DMI-6000B microscope equipped with a Yokogawa CSU-W1 spinning disk confocal unit. A 488-nm laser was used for GFP excitation and an Andor iXon Ultra DU888 EMCCD camera for signal detection.

Onset of LLPS was determined by identifying the time point of foci formation, and the number of initial foci per cell in the corresponding frames were then counted using the morphological component detection function of Mathematica (Wolfram Research). The data was plotted and analyzed with GraphPad Prism using unpaired t-tests.

### Estimation of nuclear GFP-TDP43 levels in stable cell lines

Stable HEK-293T GFP-TDP43 CTD variant cell lines (see Experimental Model and Subject Details) were seeded into eight-well ibidi µ-slides (3 × 10^4^ cells/well), allowed to adhere overnight and imaged live in L15 medium (Gibco) supplemented with 10% FBS at 37 °C using a Leica SP8 laser-scanning confocal microscope equipped with a 488-nm laser, ×63 glycerol objective (NA 1.3) and a temperature-controlled incubation chamber (Life Imaging Services) set to 37 °C. To convert fluorescence intensities into estimates of nuclear TDP43 concentrations, the microscopy setup was calibrated with a titration series of 1, 2, 5, and 10 µM GFP solutions in L15 medium. See Supplementary Fig. 8d for calibration curve.

### Purification of recombinant TDP43

The indicated TDP43 variants were appended with cleavable N-terminal His14-SUMO and C-terminal TEV-mCherry tags (Supplementary Table 2) and expressed in *E.coli* Express cells (New England Biolabs) at 20 °C in a 5-liter BioFlo110 bioreactor (New Brunswick) for 4 h after induction with 50 µM IPTG. Prior to harvesting, 1 mM PMSF and 10 mM EDTA were added to the cultures. Bacterial pellets were resuspended in IMAC buffer (20 mM Tris-HCl pH8.0, 1000 mM NaCl, 10% glycerol) and flash-frozen in liquid nitrogen. During thawing, 40 mM imidazole, 5 mM DTT and SigmaFast protease inhibitor cocktail (Sigma–Aldrich) were added. To facilitate lysis, cells were sonicated on ice. Lysates were cleared by ultra-centrifugation and TDP43 variants purified over Ni^2+^-NTA agarose beads (Gold Bio). Proteins were eluted in IMAC buffer adjusted to 400 mM imidazole and N-terminal tags cleaved-off by addition of SUMO protease. The eluates were further purified by gel filtration on a Superdex200 16/60 column (GE Healthcare) in GF buffer (40 mM HEPES pH7.4, 300 mM NaCl, 1 mM DTT). Purified TDP43 variants were concentrated using Amicon Ultracel 3 K filters (Millipore).

### In vitro TDP43 phase separation assays

Purified, full-length TDP43-TEV-mCherry variants were diluted to the indicated concentrations and adjusted to physiological conditions (20 mM HEPES pH7.4, 150 mM NaCl, 1 mM DTT). Where indicated, 10% 1,6-hexandediol (Sigma–Aldrich) or varying amounts of RNA extracted from 293 T cells using the Trizol reagent (Ambion) were additionally included. Phase separation was triggered by addition of 0.03 mg/ml TEV protease and monitored either by (i) measuring absorbance at 430 nm over time on a Synergy H1 plate reader (BioTek), (ii) imaging 30 min after TEV protease addition using a Leica SP8 laser-scanning confocal microscope equipped with a ×10 dry objective (NA 0.3) in transmitted light mode or (iii) on a 10% denaturing polyacrylamide gel after 2 h of incubation and spinning the samples at max. speed in an Eppendorf table-top microfuge for 30 min. All assays were performed at room temperature.

### Splicing reporter assays

HEK293T TDP43 knock-out cells were seeded into standard P6 tissue culture plates (at 4 × 10^5^ cells/well), allowed to adhere overnight and transfected with the indicated splicing reporter constructs (1 µg/well) using X-tremeGENE 9 (Roche). Each reporter comprised–expressed from a bidirectional promoter–the splicing module shown in Fig. 7a and a full-length TDP43 variant fused to an N-terminal BFP tag. Twenty four hour after transfection, cells were harvested by trypsinization for flow cytometry or using Trizol reagent (Ambion) for RNA extraction.

All flow cytometry-based splicing assays were analyzed with a Sony SH800 flow cytometer. Instruments parameter settings were adjusted following the compensation guidelines using cells either expressing BFP, GFP, or mCherry, respectively. Data for at least 40,000 BFP-positive single cells per TDP43 variant were collected and analyzed with custom pipelines available on Github (https://github.com/RohatgiLab/TDP43-analysis) to calculate and plot the mCherry/GFP fluorescence intensity ratio and BFP levels on a single-cell level. Gate boundaries are specified in the code and a graphical representation of the applied gating strategy is shown in Supplementary Fig. 12.

To validate and corroborate the flow cytometry-based splicing measurements, total RNA was extracted using the Trizol reagent (Ambion) according to the manufacturer’s instructions, transcribed into cDNA using the iScript kit (Bio-Rad) and analyzed by analytical RT-PCR using the GoTaq Green kit (Promega) with primers 1171 and 1174 (see Supplementary Table 1). PCR products were separated by electrophoresis on 1.5–2% agarose gels and documented with an AlphaImager EC system (Alpha Innotech).

### Splicing assay for endogenous TDP43 target RNAs

To test for splicing defects in the indicated endogenous TDP43 targets, total RNA was first extracted from >1.0 × 10^6^ cells using Trizol reagent (Ambion) following the manufacturer’s instructions, then transcribed into cDNA using the iScript kit (Bio-Rad) and subsequently analyzed using the GoTaq Green kit (Promega) for analytical RT-PCRs with the primers specified in Supplementary Table 1. PCR products were electrophoresed on 1.5–2% agarose gels and documented with an AlphaImager EC system (Alpha Innotech).

### Reporting summary

Further information on research design is available in the Nature Research Reporting Summary linked to this article.

## Supplementary information

Supplementary Information

Reporting Summary

Source Data

## Data Availability

The source data underlying Figs. 3b, 3d, 7d and 8, as well as Supplementary Figs. 3b, 5b, 5c and 6b are provided as a Source Data file. The raw data generated in this study is available from the corresponding authors upon reasonable request.

## Code availability

All custom pipelines used in this study are available on Github (https://github.com/RohatgiLab/TDP43-analysis).
