# Supplementary material for: Phase separation-deficient TDP43 remains functional in splicing
Source: Nat Commun. 2019 Oct 25;10:4890. doi: 10.1038/s41467-019-12740-2 (PMC6814767; doi:10.1038/s41467-019-12740-2)
Supplement: Supplementary file 1 — Supplementary Information [file 41467_2019_12740_MOESM1_ESM.pdf]

## Supplementary Information

# Phase separation-deficient TDP43 remains functional in splicing

Hermann Broder Schmidt et al.

Supplementary Table 1. Oligonucleotides used in this study.

| Sequence | Source | Identifier |
|---|---|---|
| 5'-caccgagatgctggctggggaaatc-3' | This study | 704 TARDBP-ko-1-fwd |
| 5'-aaacgatttccccagccagcatctc-3' | This study | 705 TARDBP-ko-1-rev |
| 5'-caccgaacatccgatttaatagtgt-3' | This study and Ref #1 | 706 TARDBP-ko-2-fwd |
| 5'-aaacacactattaaatcggatgttc-3' | This study and Ref #1 | 707 TARDBP-ko-2-rev |
| 5'-tggacgatggtgtgactgcaa-3' | Ref #2 | 812 HsTARDBP qPCR fwd |
| 5'-agagaagaactcccgcagctca-3' | Ref #2 | 813 HsTARDBP qPCR rev |
| 5'-gttcatgcgcttcaaggtg-3' | This study | 1171 Cryptic Exon fwd |
| 5'-ttggtcaccttcagcttgg-3' | This study | 1174 Cryptic Exon rev |
| 5'-ggaccagcgttccctgtgc-3' | This study | 1019 HsATG4B fwd |
| 5'-caccaatcattgaagtcatc-3' | This study | 1020 HsATG4B rev |
| 5'-tccttgggttggacaagaac-3' | This study | 1311 HsDNAJC5 fwd |
| 5'-ttgaacccgtcagtgtggta-3' | This study | 1312 HsDNAJC5 rev |
| 5'-ggaggaaaatttgataagcatgag-3' | This study | 1491 HsGPSM2 fwd |
| 5'-aactgcatccctgaagttgc-3' | This study | 1492 HsGPSM2 rev |
| 5'-gcggcgcagatggagcag-3' | This study | 1021 HsITPR3 fwd |
| 5'-ccgaggtggcagactggc-3' | This study | 1022 HsITPR3 rev |
| 5'-cgagatccctccaaaatcaa-3' | This study | 1442 HsGAPDH fwd |
| 5'-aggggtctacatggcaactg-3' | This study | 1443 HsGAPDH rev |

Supplementary Table 2. Plasmids used in this study.

| Plasmid | Reference | Accession Number |
|---|---|---|
| pHBS838 TDP43$_{RRM-GFP}$ WT | Ref #3 | Addgene #98249 |
| pHBS941 TDP43$_{RRM-GFP}$ Φ-S | This study | Addgene #121992 |
| pHBS1383 TDP43$_{RRM-GFP}$ FYW-S | This study | Addgene #118791 |
| pHBS1384 TDP43$_{RRM-GFP}$ FYW-L | This study | Addgene #118793 |
| pHBS931 TDP43$_{RRM-GFP}$ F-S | This study | Addgene #107799 |
| pHBS1385 TDP43$_{RRM-GFP}$ VLIM-S | This study | Addgene #118792 |
| pHBS1386 TDP43$_{RRM-GFP}$ VLIM-F | This study | Addgene #118794 |
| pHBS935 TDP43$_{RRM-GFP}$ M-S | This study | Addgene #107800 |
| pHBS934 TDP43$_{RRM-GFP}$ M-V | This study | Addgene #107801 |
| pHBS1037 TDP43$_{RRM-GFP}$ 6xΦ | This study | Addgene #107804 |
| pHBS1038 TDP43$_{RRM-GFP}$ 4xΦ | This study | Addgene #107805 |
| pHBS1039 TDP43$_{RRM-GFP}$ 2xΦ | This study | Addgene #107806 |
| pHBS1387 TDP43$_{RRM-GFP}$ KRED-S | This study | Addgene #118795 |
| pHBS1052 TDP43$_{RRM-GFP}$ R-K | This study | Addgene #107856 |
| pHBS1388 TDP43$_{RRM-GFP}$ K-R | This study | Addgene #118796 |
| pHBS1256 TDP43$_{RRM-GFP}$ W385G | This study | Addgene #107807 |
| pHBS1291 TDP43$_{RRM-GFP}$ G368W | This study | Addgene #107837 |
| pHBS1292 TDP43$_{RRM-GFP}$ G368W+W385G | This study | Addgene #107838 |
| pHBS966 TDP43$_{RRM-GFP}$ G309F | This study | Addgene #107822 |
| pHBS968 TDP43$_{RRM-GFP}$ G309S | This study | Addgene #107824 |
| pHBS1280 TDP43$_{RRM-GFP}$ G348F | This study | Addgene #107830 |
| pHBS1279 TDP43$_{RRM-GFP}$ G348V | This study | Addgene #107831 |
| pHBS834 H14-SUMO-TDP43 WT-TEV-mCherry | This study | Addgene #133320 |
| pHBS1551 H14-SUMO-TDP43 F-S-TEV-mCherry | This study | Addgene #133321 |

| | | |
|---|---|---|
| pHBS1552 H14-SUMO-TDP43 FYW-S-TEV-mCherry | This study | Addgene #133322 |
| pHBS1147 GFP-TDP43 | This study | Addgene #118797 |
| pHBS1410 GFP-TDP43 F-S | This study | Addgene #118799 |
| pHBS1411 GFP-TDP43 FYW-S | This study | Addgene #118800 |
| pHBS1412 GFP-TDP43 FYW-L | This study | Addgene #118801 |
| pHBS1415 GFP-TDP43 VLIM-S | This study | Addgene #133323 |
| pHBS1413 GFP-TDP43 VLIM-F | This study | Addgene #118802 |
| pHBS1389 IBB-GFP-mCherry3E | This study | Addgene #118803 |
| pHBS1500 [IBB-GFP-mCherry3E]-[BFP-TDP43 ΔNTD] | This study | Addgene #133324 |
| pHBS1501 [IBB-GFP-mCherry3E]-[BFP-TDP43 ΔRRM] | This study | Addgene #133325 |
| pHBS1502 [IBB-GFP-mCherry3E]-[BFP-TDP43 ΔCTD] | This study | Addgene #133326 |
| pHBS1503 [IBB-GFP-mCherry3E]-[BFP-TDP43 WT] | This study | Addgene #133327 |
| pHBS1504 [IBB-GFP-mCherry3E]-[BFP-TDP43 F-S] | This study | Addgene #133328 |
| pHBS1505 [IBB-GFP-mCherry3E]-[BFP-TDP43 FYW-S] | This study | Addgene #133329 |
| pHBS1506 [IBB-GFP-mCherry3E]-[BFP-TDP43 FYW-L] | This study | Addgene #133330 |
| pHBS1507 [IBB-GFP-mCherry 3E]-[BFP-TDP43 VLIM-S] | This study | Addgene #133331 |
| pHBS1508 [IBB-GFP-mCherry 3E]-[BFP-TDP43 VLIM-F] | This study | Addgene #133332 |
| pHBS1527 [IBB-GFP-mCherry3E]-[BFP-TDP43 W-S] | This study | Addgene #133333 |
| pHBS952 TARDBP43-Exon1-ko-sgRNA1-SpCas9 | This study | Addgene #107857 |
| pHBS953 TARDBP43-Exon2-ko-sgRNA2-SpCas9 | This study | Addgene #107858 |
| pMD2.G | Didier Trono Lab | Addgene #12259 |
| psPAX2 | Didier Trono Lab | Addgene #12260 |

**Supplementary Table 3.** Sequences of compositional CTD mutants.

Legend:
**Hydrophobic residue**
Conserved region (not mutated)

**>WT**
KHNSNRQLERSGRFGGNPGGFGNQGGFGNSRGGGAGLGNNQGSNMGGGMNFGAFSINPAMMAAAQAALQSSWGMMGM
LASQQNQSGPSGNNQNQGNMQREPNQAFGSGNNSYSGSNSGAAIGWGSASNAGSGSGFNGGFGSSMDSKSSGWGM.

**>Φ-S**
KHNSNRQSERSGRSGGNPGGSGNQGGSGNSRGGGAGSGNNQGSNSGGGSNSGASSSNPAMMAAAQAALQSSWGMMGM
LASQQNQSGPSGNNQNQGNSQREPNQASGSGNNSSSGSNSGAASGSGSASNAGSGSGSNGGSGSSSDSKSSGSGS.

**>F-S**
KHNSNRQLERSGRSGGNPGGSGNQGGSGNSRGGGAGLGNNQGSNMGGGMNSGASSINPAMMAAAQAALQSSWGMMGM
LASQQNQSGPSGNNQNQGNMQREPNQASGSGNNSYSGSNSGAAIGWGSASNAGSGSGSNGGSGSSMDSKSSGWGM.

**>FYW-S**
KHNSNRQLERSGRSGGNPGGSGNQGGSGNSRGGGAGLGNNQGSNMGGGMNSGASSINPAMMAAAQAALQSSWGMMGM
LASQQNQSGPSGNNQNQGNMQREPNQASGSGNNSSSGSNSGAAIGSGSASNAGSGSGSNGGSGSSMDSKSSGSGM.

**>VLIM-S**
KHNSNRQSERSGRFGGNPGGFGNQGGFGNSRGGGAGSGNNQGSNSGGGSNFGAFSSNPAMMAAAQAALQSSWGMMGM
LASQQNQSGPSGNNQNQGNSQREPNQAFGSGNNSYSGSNSGAASGWGSASNAGSGSGFNGGFGSSSDSKSSGWGS.

**>FYW-L**
KHNSNRQLERSGRLGGNPGGLGNQGGLGNSRGGGAGLGNNQGSNMGGGMNLGALSINPAMMAAAQAALQSSWGMMGM
LASQQNQSGPSGNNQNQGNMQREPNQALGSGNNSLSGSNSGAAIGLGSASNAGSGSGLNGGLGSSMDSKSSGLGM.

**>VLIM-F**
KHNSNRQFERSGRFGGNPGGFGNQGGFGNSRGGGAGFGNNQGSNFGGGFNFGAFSFNPAMMAAAQAALQSSWGMMGM
LASQQNQSGPSGNNQNQGNFQREPNQAFGSGNNSYSGSNSGAAFGWGSASNAGSGSGFNGGFGSSFDSKSSGWGF.

**>R-K**
KHNSNKQLEKSGKFGGNPGGFGNQGGFGNSKGGGAGLGNNQGSNMGGGMNFGAFSINPAMMAAAQAALQSSWGMMGM
LASQQNQSGPSGNNQNQGNMQKEPNQAFGSGNNSYSGSNSGAAIGWGSASNAGSGSGFNGGFGSSMDSKSSGWGM.

**>K-R**
RHNSNRQLERSGRFGGNPGGFGNQGGFGNSRGGGAGLGNNQGSNMGGGMNFGAFSINPAMMAAAQAALQSSWGMMGM
LASQQNQSGPSGNNQNQGNMQREPNQAFGSGNNSYSGSNSGAAIGWGSASNAGSGSGFNGGFGSSMDSRSSGWGM.

**>KRED-S**
SHNSNSQLSSSGSFGGNPGGFGNQGGFGNSSGGGAGLGNNQGSNMGGGMNFGAFSINPAMMAAAQAALQSSWGMMGM
LASQQNQSGPSGNNQNQGNMQSSPNQAFGSGNNSYSGSNSGAAIGWGSASNAGSGSGFNGGFGSSMSSSSSGWGM.

**>F-Y**
KHNSNRQLERSGRYGGNPGGYGNQGGYGNSRGGGAGLGNNQGSNMGGGMNYGAYSINPAMMAAAQAALQSSWGMMGM
LASQQNQSGPSGNNQNQGNMQREPNQAYGSGNNSYSGSNSGAAIGWGSASNAGSGSGYNGGYGSSMDSKSSGWGM.

**>M-S**
KHNSNRQLERSGRFGGNPGGFGNQGGFGNSRGGGAGLGNNQGSNSGGGSNFGAFSINPAMMAAAQAALQSSWGMMGM
LASQQNQSGPSGNNQNQGNSQREPNQAFGSGNNSYSGSNSGAAIGWGSASNAGSGSGFNGGFGSSSDSKSSGWGS.

**>M-V**
KHNSNRQLERSGRFGGNPGGFGNQGGFGNSRGGGAGLGNNQGSNVGGGVNFGAFSINPAMMAAAQAALQSSWGMMGM
LASQQNQSGPSGNNQNQGNVQREPNQAFGSGNNSYSGSNSGAAIGWGSASNAGSGSGFNGGFGSSVDSKSSGWGV.

**>W-S**
KHNSNRQLERSGRFGGNPGGFGNQGGFGNSRGGGAGLGNNQGSNMGGGMNFGAFSINPAMMAAAQAALQSSWGMMGM
LASQQNQSGPSGNNQNQGNMQREPNQAFGSGNNSYSGSNSGAAIGSGSASNAGSGSGFNGGFGSSMDSKSSGSGM.

**>6x Φ clusters**
KHNSNRQLERSGRGNPFGGGFGNQNSRGGGAGGGFGLGNNQGSNMGGGMNAFGFSINPAMMAAAQAALQSSWGMMGM
LASQQNQSGPSGNNQNQGNMQREPNQAFGYSSGNNSGSNSGAAIGWGSASNAGSGSNGFGGFGGWGMSSMDSKSS.

**>4x Φ clusters**
KHNSNRQLERSGRGNPFGGGFGGGFGNQNSRGGGAGNNQGSNLGMGFGFSGGMNAINPAMMAAAQAALQSSWGMMGM
LASQQNQSGPSGNNQNQGNMQREPNQASGNNSFGYSIGWGGSNSGAASASNAGSGSNGFGGFGGWGMSSMDSKSS.

**>2x Φ clusters**
KHNSNRQLERSGRGNPNQNSRGGFGGGFGGGFGLGMGFGFSGAGNNQGSNGGMNAINPAMMAAAQAALQSSWGMMGM
LASQQNQSGPSGNNQNQGNMQREPNQASGNNSFGYSIGWGGFGGFGGWGMSGNSGAASASNAGSGSNSMDSKSS.

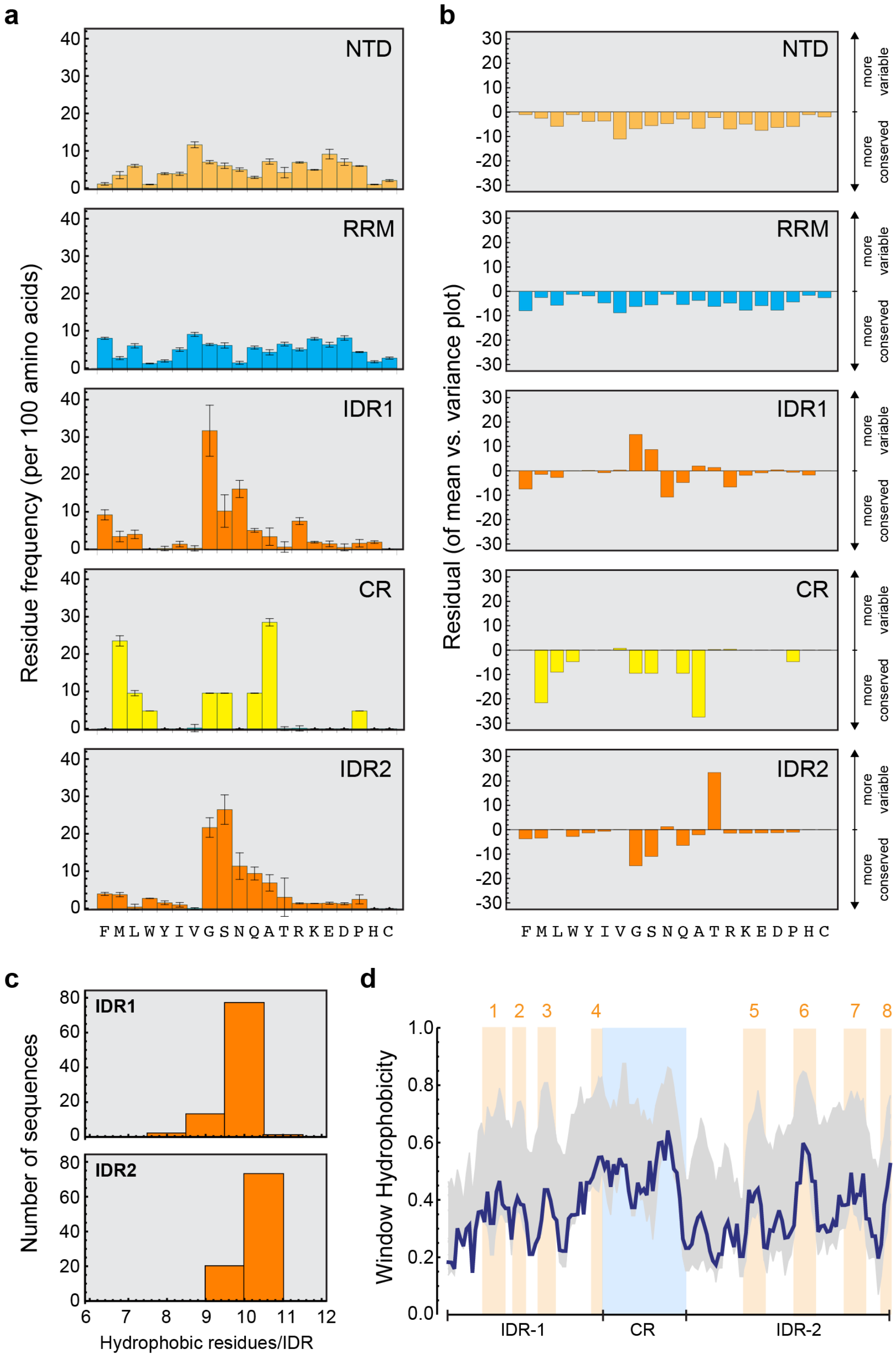


**Supplementary Figure 1. Compilation and analysis of a vertebrate TDP43 homolog database.**

(**a**) Comparison of the amino acid composition of the TDP43 IDRs and CRs to the structured NTDs and RRM domains. Shown are mean amino acid frequencies per 100 residues ± standard deviation (n = 93).

(**b**) Comparison of residuals from the mean frequency vs. variance regression plots for each standard amino acid to assess residue conservation in the NTDs, RRMs, IDRs and CRs of 93 vertebrate TDP43 homologs. A negative residual indicates low variance in the abundance of a

given amino acid in the indicated TDP43 domains and suggests that this residue is conserved. Conversely, a positive residual indicates that the abundance of a given amino acid is variable and suggests that it is not conserved.

(**c**) Histograms showing the variation in the number of hydrophobic residues (V, L, I, M, F, Y, W) in the IDRs of 93 vertebrate TDP43 CTDs.

(**d**) Comparison of the CTD hydrophobicity profile using different hydrophobicity scales. Hydrophobicity was calculated in a sliding window of 5 amino acids with the ProtScale tool from ExPASy using different hydrophobicity scales (Abraham & Leo, Black, Cothia, Eisenberg et al., Fauchère & Pliska, Janin, Kyte & Doolittle, Manavalan et al., Miyazawa et al., Rao & Argos, Roseman, Tanford, Welling et al. and Wolfenden et al.). Blue trace: CTD hydrophobicity according to the scale of Fauchère & Pliska, used throughout the main figures in the manuscript. Light gray area: deviation in hydrophobicity profile predictions across all scales. Yellow shading highlights major hydrophobic clusters. Results were re-scaled to range between 0 and 1 for comparison.

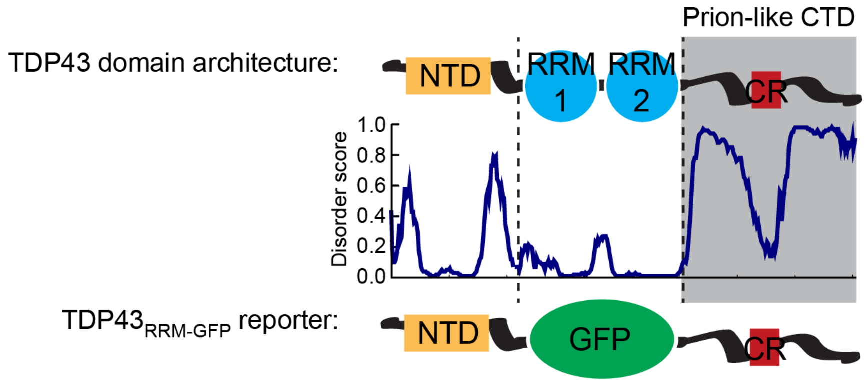


**Supplementary Figure 2. Design of the TDP43$_{\text{RRM-GFP}}$ reporter.**
Illustration of the TDP43$_{\text{RRM-GFP}}$ reporter design. Note that replacing the RRM domains with GFP leaves the overall domain architecture of TDP43 intact.

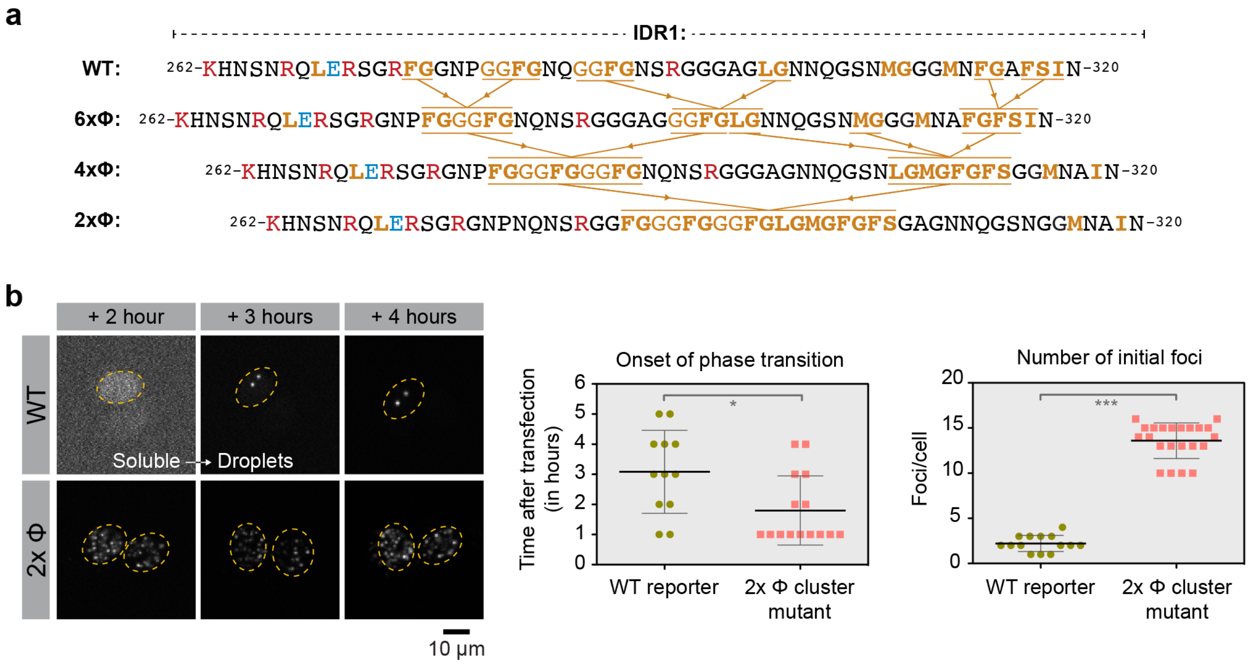


**Supplementary Figure 3. Design of CTD mutants with altered spacing between hydrophobic clusters.**

(**a**) The spacing between hydrophobic clusters was changed by sliding adjacent sets of clusters together, illustrated for how the WT sequence was changed to the 6xΦ, 4xΦ and 2xΦ variants in IDR1. Hydrophobic motifs are in orange, positively-charged residues in red, and negatively-charged residues in blue.

(**b**) Time course of condensate formation by the WT and 2xΦ variants of $TDP43_{RRM-GFP}$. Representative images of live cells expressing the WT or 2xΦ variants at the indicated time points after transient transfection and scatter plots comparing the mean time of onset for focus formation ($p = 0.01$, Student's t-test) and the mean number of foci per cell in the first frame after phase separation ($p < 0.0001$, Student's t-test). Black horizontal lines indicate means and gray error bars standard deviation ($n \geq 12$ cells).

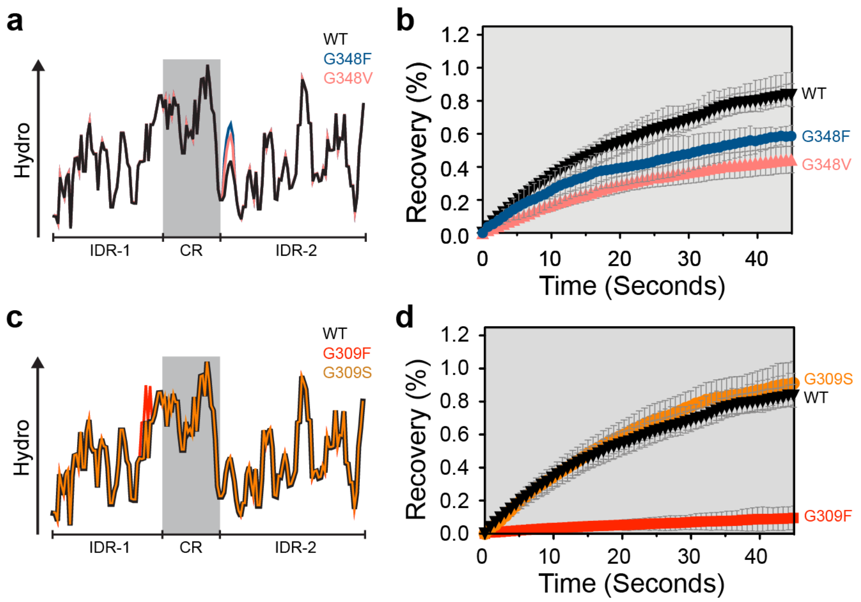


**Supplementary Figure 4. Phase behavior of TDP43$_{\text{RRM-GFP}}$ droplets carrying point mutations in IDR1 and IDR2.**

(**a**) The effects of the G348F and G348V mutations on local hydrophobicity predict that both mutations reduce TDP43 phase dynamics.

(**b**) Half-bleach FRAP experiments of G348V and G348F mutant TDP43 droplets show that both mutations reduced fluidity. Plot markers represent mean values and error bars standard deviation ($n \geq 20$).

(**c**) The effects of the G309F and G309S mutations on local hydrophobicity predict that only the G309F mutation should alter TDP43 phase dynamics.

(**d**) Half-bleach FRAP experiments revealed that the G309F mutation reduced droplet fluidity, while the G309S mutation had no effect. Plot markers represent mean values and error bars standard deviation ($n \geq 6$).

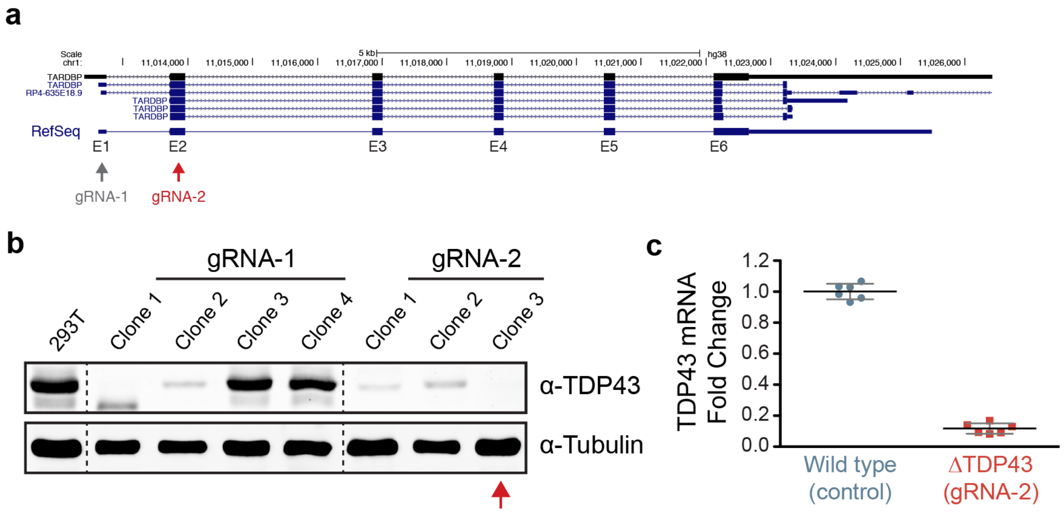


**Supplementary Figure 5. Generation of TDP43-/- HEK-293T cells.**
(**a**) Structure of the *TARDBP* locus (from the UCSC genome browser) showing the exons targeted by two independent sgRNAs.
(**b**) Immunoblot showing TDP43 and α-tubulin (loading control) protein levels in parent HEK-293T cells and clonal derivatives after transient expression of Cas9 and the indicated sgRNAs. The red arrow indicates the clone used to make stable cell lines (clone 3, sgRNA-2).
(**c**) TDP43 mRNA levels were measured using qRT-PCR in HEK-293T cells (WT control) and a clonal TDP43-/- HEK-293T cell line (clone 3, sgRNA-2 in B). Scatter plot showing means (black horizontal line) and standard deviation (gray error bars). Data from two biological replicates with three technical replicates each.

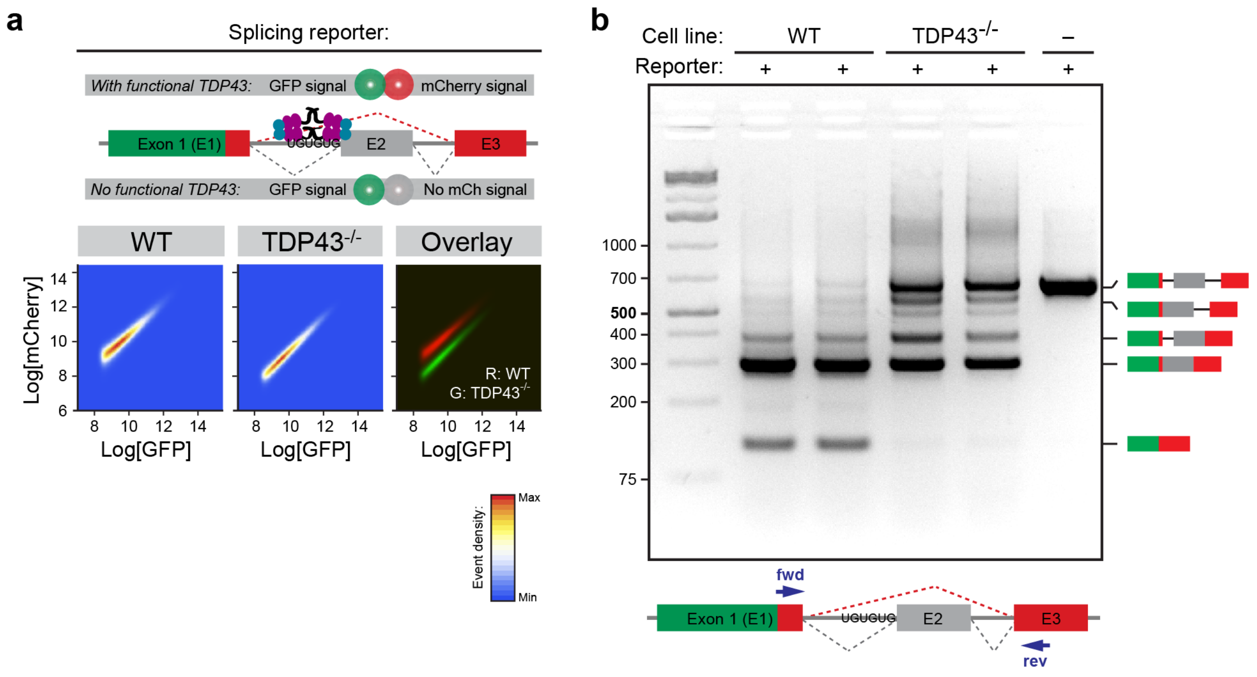


**Supplementary Figure 6. Characterization of the TDP43-dependent fluorescent splicing reporter in WT and TDP43-/- HEK-293T cells.**

(**a**) Flow cytometry plots showing the distribution of GFP and mCherry fluorescence signals from cells transiently transfected with the splicing reporter shown above. Retention of Exon 2 (E2) will reduce mCherry fluorescence but will leave the GFP signal intact, reducing the mCherry/GFP ratio. Data from WT and *TDP43-/-* cells (left and middle panels) are color-coded as heat maps representing cell density and are overlaid in the right panel for comparison (WT cells in red and *TDP43-/-* cells in green). For each value of GFP fluorescence, the mCherry fluorescence is lower in the *TDP43-/-* cells.

(**b**) RT-PCR analysis to determine the splicing pattern of the reporter in WT and TDP43-/- HEK-293T cells (clone 3, sgRNA2). The cartoon below the gel shows the annealing positions of the primers used in the assay and cartoons to the right of the gel illustrate the structure of various splicing products and intermediates.

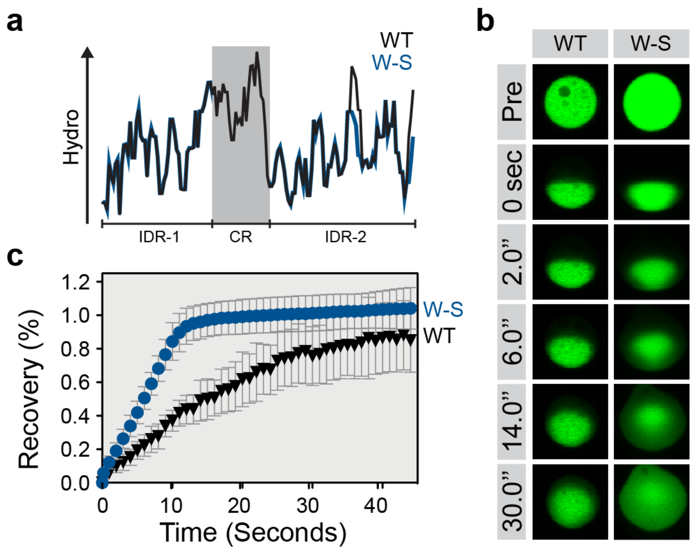


**Supplementary Figure 7. Phase behavior of $TDP43_{RRM\text{-}GFP}$ droplets with W-S mutations in IDR2.**

(**a**) Effect of the W-S mutation on local hydrophobicity.

(**b**) Half-bleach FRAP experiments of W-S mutant TDP43 droplets show enhanced fluidity.

(**c**) Plots showing the time-dependent, normalized fluorescence recovery of the half-bleach experiments in (b). Plot markers represent mean values and error bars standard deviation (n ≥ 5).

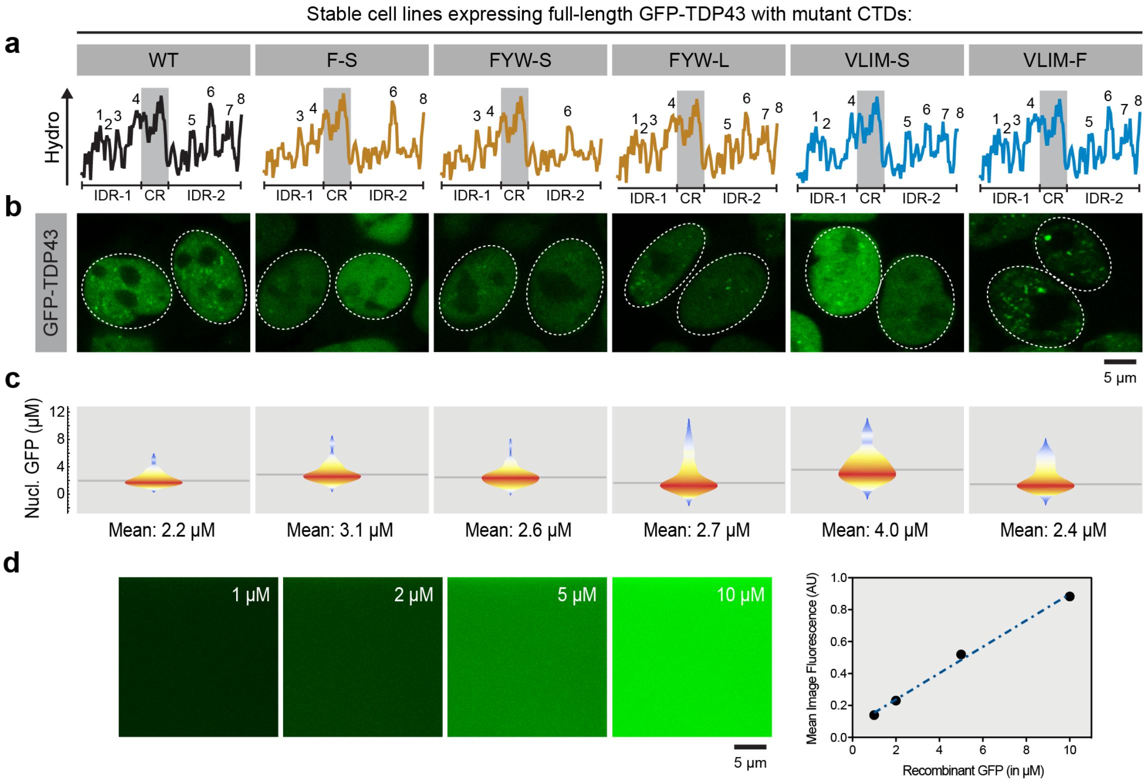


**Supplementary Figure 8. Generation of stable GFP-TDP43 add-back cell lines.**
(**a**) Hydrophobicity plots for the indicated CTD variants, with numbers highlighting the major hydrophobic clusters.
(**b**) Representative confocal images (63x) showing nuclear GFP-TDP43 signals after stable add-back into TDP43-/- HEK-293T cells. Dashed white lines denotes outline of nuclei.
(**c**) Violin plots showing the distribution of estimated nuclear GFP-TDP43 concentrations in the indicated cell lines. At least 40 nuclei per cell line were quantified.
(**d**) Titration series of purified GFP at the indicated concentrations (left) and standard curve to convert fluorescence signals into concentrations (right).

```
KHNSNRQLER SGRFGGNPGG FGNQGGFGNS
RGGGAGLGNN QGSNMGGGMN FGAFSINpam
maaaqaalqs swgmmgmlas qqnqsgpSGN
NQNQGNMQRE PNQAFGSGNN SYSGSNSGAA
IGWGSASNAG SGSGFNGGFG SSMDSKSSGW
GM
```

Predicted LARKs

Conserved region

**Supplementary Figure 9. LARKs are confined to IDR1 of TDP43.**
Grey shading indicates the localization of the predicted LARKs in the TDP43 IDRs. The conserved region (lower case letters) is shaded in red. See Discussion for details.

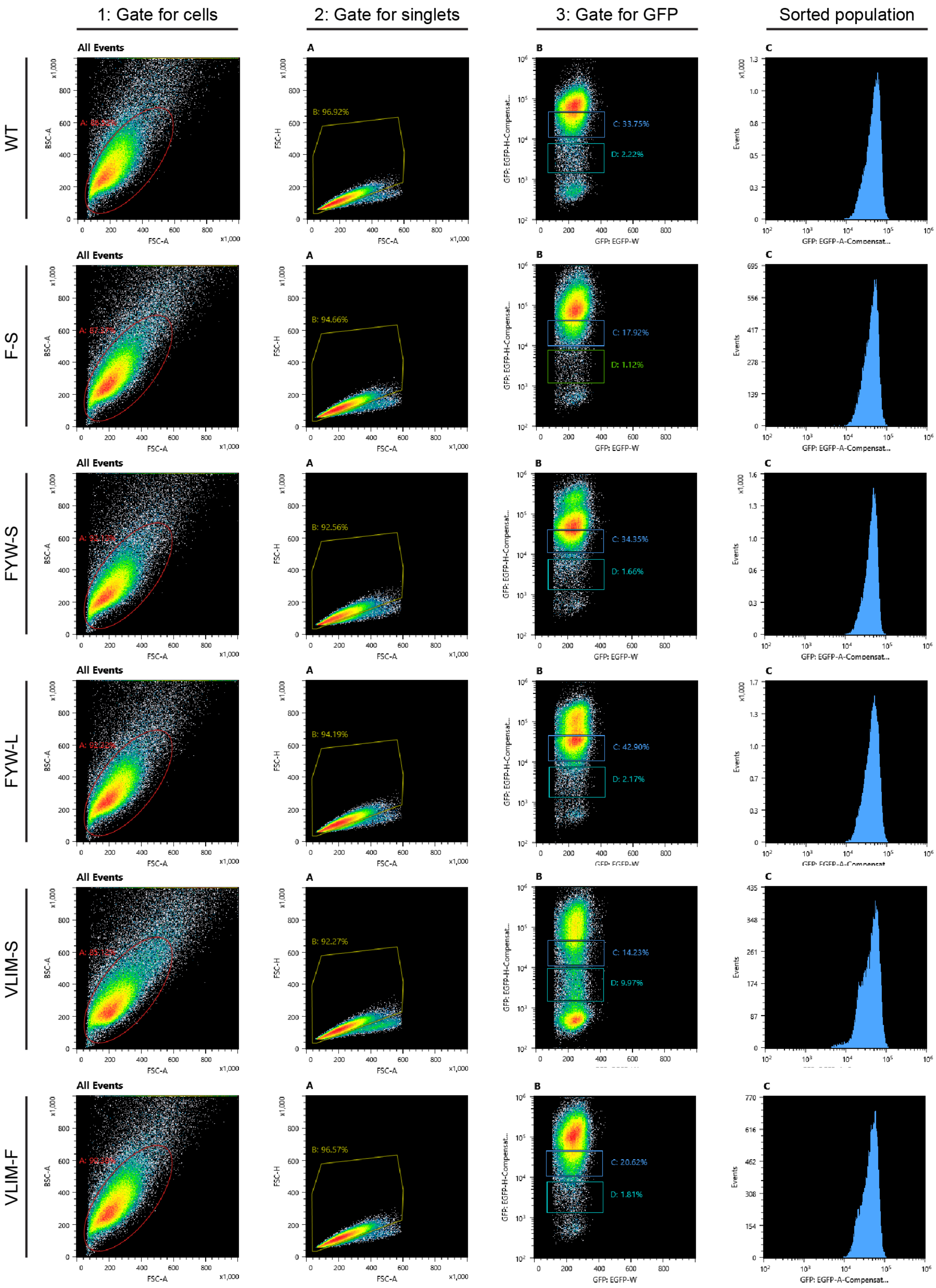
1: Gate for cells
2: Gate for singlets
3: Gate for GFP
Sorted population
WT
F-S
FYW-S
FYW-L
VLIM-S
VLIM-F
All Events
A
B
C
BSC-A
FSC-A
FSC-H
x1,000
GFP: EGFP-H-Compensat...
GFP: EGFP-W
GFP: EGFP-A-Compensat...
Events
B: 96.92%
C: 33.75%
D: 2.22%
B: 94.66%
C: 17.92%
D: 1.12%
B: 92.56%
C: 34.35%
D: 1.66%
B: 94.19%
C: 42.90%
D: 2.17%
B: 92.27%
C: 14.23%
D: 9.97%
B: 96.57%
C: 20.62%
D: 1.81%

**Supplementary Figure 10. Gating strategy used to sort GFP-TDP43 variant cell lines.** Total events were first gated for the population that corresponds to cells (gate A), then for singlets (gate B) and eventually sorted for cells with comparable GFP signals (gate C). Histograms show the total GFP signal of the sorted populations that constitute the cell lines used in Figure 8 and Supplementary Figure 8. See Methods for details on cell line generation.

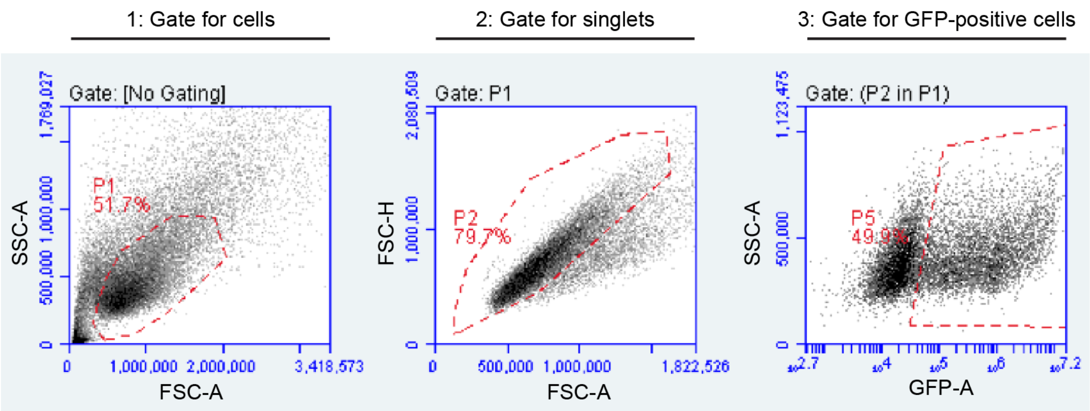


**Supplementary Figure 11. Gating strategy used to compare total GFP levels in cells transfected with TDP43$_{RRM-GFP}$ variants.**

For the analysis of total GFP levels in Figures 2 and 5, total events were first gated for the population that corresponds to cells (gate P1), then for singlets (gate P2) and eventually GFP-positive cells (gate P5). At least 10,000 events were collected in gate P2 for all TDP43$_{RRM-GFP}$ variants analyzed. Routine transfection efficiencies were 40-60%. See Methods for details.

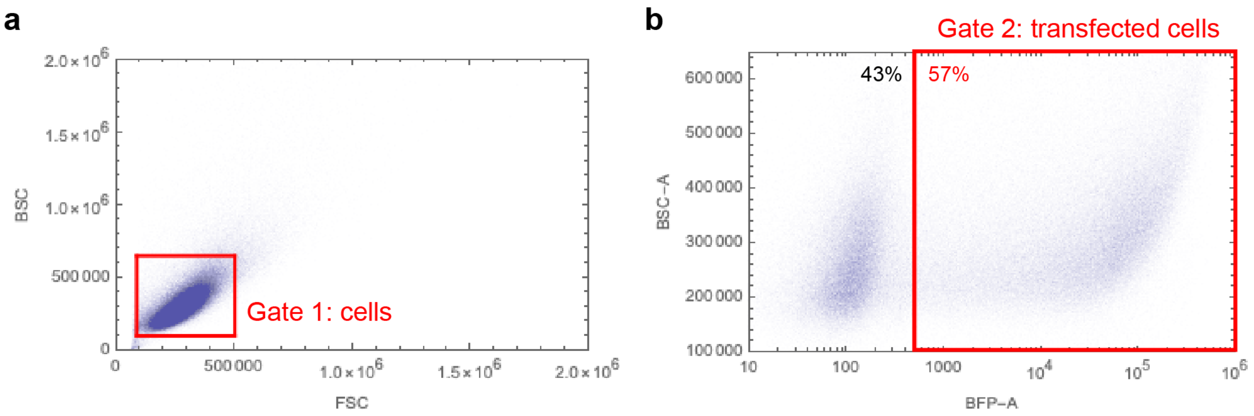


**Supplementary Figure 12. Gating strategy used in flow cytometry analysis of TDP43 splicing function.**

(**a**) Total events were first gated for the population that corresponds to cells and 100,000 gated events recorded for each analysis run.

(**b**) The cell population form (a) was then further gated for transfected cells based on BFP fluorescence. Standard transfection efficiencies in all experiments were 40-60%. See methods for details and source code availability.

## Supplementary References

1. Shalem, O. et al. Genome-scale CRISPR-Cas9 knockout screening in human cells. *Science* **343**, 84-87 (2014).
2. Prudencio, M. et al. Misregulation of human sortilin splicing leads to the generation of a nonfunctional progranulin receptor. *Proc Natl Acad Sci U S A* **109**, 21510-21515 (2012).
3. Schmidt, H. B. & Rohatgi, R. In Vivo Formation of Vacuolated Multi-phase Compartments Lacking Membranes. *Cell Rep* **16**, 1228-1236 (2016).
