# Supplementary material for: Phase separation-deficient TDP43 remains functional in splicing
Source: Nat Commun. 2019 Oct 25;10:4890. doi: 10.1038/s41467-019-12740-2 (PMC6814767; doi:10.1038/s41467-019-12740-2)
Supplement: Supplementary file 2 — Reporting Summary [file 41467_2019_12740_MOESM2_ESM.pdf]

nature research

Corresponding author(s): Rajat Rohatgi, Broder Schmidt

Last updated by author(s): Sep 23, 2019

Reporting Summary

Nature Research wishes to improve the reproducibility of the work that we publish. This form provides structure for consistency and transparency in reporting. For further information on Nature Research policies, see Authors & Referees and the Editorial Policy Checklist.

Statistics

For all statistical analyses, confirm that the following items are present in the figure legend, table legend, main text, or Methods section.

n/a	Confirmed	
☐	☒	The exact sample size (n) for each experimental group/condition, given as a discrete number and unit of measurement
☐	☒	A statement on whether measurements were taken from distinct samples or whether the same sample was measured repeatedly
☐	☒	The statistical test(s) used AND whether they are one- or two-sided<br>*Only common tests should be described solely by name; describe more complex techniques in the Methods section.*
☒	☐	A description of all covariates tested
☒	☐	A description of any assumptions or corrections, such as tests of normality and adjustment for multiple comparisons
☐	☒	A full description of the statistical parameters including central tendency (e.g. means) or other basic estimates (e.g. regression coefficient) AND variation (e.g. standard deviation) or associated estimates of uncertainty (e.g. confidence intervals)
☐	☒	For null hypothesis testing, the test statistic (e.g. F, t, r) with confidence intervals, effect sizes, degrees of freedom and P value noted<br>*Give P values as exact values whenever suitable.*
☒	☐	For Bayesian analysis, information on the choice of priors and Markov chain Monte Carlo settings
☒	☐	For hierarchical and complex designs, identification of the appropriate level for tests and full reporting of outcomes
☒	☐	Estimates of effect sizes (e.g. Cohen's d, Pearson's r), indicating how they were calculated

Our web collection on statistics for biologists contains articles on many of the points above.

Software and code

Policy information about availability of computer code

Data collection	Leica Application Suite X, Sony SH800 flow cytometer software
Data analysis	Wolfram Mathematica 11 (custom code available on Github: https://github.com/RohatgiLab/TDP43-analysis), Graphpad Prism 5, Microsoft Excel, Leica Application Suite X

For manuscripts utilizing custom algorithms or software that are central to the research but not yet described in published literature, software must be made available to editors/reviewers. We strongly encourage code deposition in a community repository (e.g. GitHub). See the Nature Research guidelines for submitting code & software for further information.

Data

Policy information about availability of data

All manuscripts must include a data availability statement. This statement should provide the following information, where applicable:

- Accession codes, unique identifiers, or web links for publicly available datasets
- A list of figures that have associated raw data
- A description of any restrictions on data availability

The datasets generated during and/or analyzed during the current study are available from the corresponding author on reasonable request.

Field-specific reporting

Please select the one below that is the best fit for your research. If you are not sure, read the appropriate sections before making your selection.

☒ Life sciences ☐ Behavioural & social sciences ☐ Ecological, evolutionary & environmental sciences

Life sciences study design

All studies must disclose on these points even when the disclosure is negative.

Sample size	No sample size calculation was performed; sample size was chosen such that statistical significance could be confidently established.
Data exclusions	No data were excluded from the analyses.
Replication	All attempts at replication were successful.
Randomization	Not applicable, there was no experimental group allocation.
Blinding	Not applicable, there was no experimental group allocation.

Reporting for specific materials, systems and methods

We require information from authors about some types of materials, experimental systems and methods used in many studies. Here, indicate whether each material, system or method listed is relevant to your study. If you are not sure if a list item applies to your research, read the appropriate section before selecting a response.

Materials & experimental systems

n/a	Involved in the study	
☐	☒	Antibodies
☐	☒	Eukaryotic cell lines
☒	☐	Palaeontology
☒	☐	Animals and other organisms
☒	☐	Human research participants
☒	☐	Clinical data

Methods

n/a	Involved in the study	
☒	☐	ChIP-seq
☐	☒	Flow cytometry
☒	☐	MRI-based neuroimaging

Antibodies

Antibodies used	Rabbit polyclonal anti-TDP43 Proteintech Cat# 10782-2-AP<br>Mouse monoclonal anti-alpha-Tubulin Sigma-Aldrich Cat# T6199<br>IRDye 680LT Donkey anti-Mouse IgG (H+L) Li-Cor Cat# 926-68022<br>IRDye 800CW Donkey anti-Rabbit IgG (H+L) Li-Cor Cat# 926-32213
Validation	All antibodies were validated by the manufacturers. We validated the anti-TDP43 primary antibody again by showing that the putative anti-TDP43 signal seen in immunoblots is abolished when TARDBP is knocked-out.

Eukaryotic cell lines

Policy information about cell lines

Cell line source(s)	Human HEK-293T cells (ATCC CRL-3216)
Authentication	HEK-293T cells were authenticated by ATCC.
Mycoplasma contamination	The HEK-293T parent cell line from which all other cell lines in the study were derived tested negative for mycoplasma contamination.
Commonly misidentified lines (See ICLAC register)	No commonly misidentified cell lines were used.

Flow Cytometry

Plots

Confirm that:

☒ The axis labels state the marker and fluorochrome used (e.g. CD4-FITC).

☒ The axis scales are clearly visible. Include numbers along axes only for bottom left plot of group (a 'group' is an analysis of identical markers).

☒ All plots are contour plots with outliers or pseudocolor plots.

☒ A numerical value for number of cells or percentage (with statistics) is provided.

Methodology

Sample preparation	The indicated HEK-293T cells were transfected with splicing reporter constructs as described in the Methods. Cells were detached from tissue culture vessels using trypsin-EDTA. An equal volume of culture medium was added to inactivate the trypsin, cells were filtered through a cell strainer and immediately analyzed with a flow cytometer.
Instrument	Sony SH800 flow cytometer
Software	The Sony SH800 flow cytometer operating software was used for data collection and custom Mathematica analysis pipelines (available at https://github.com/RohatgiLab/TDP43-analysis) were used for data analysis.
Cell population abundance	The standard transfection efficiency achieved was 40-50%. Only transfected cells were used for analysis.
Gating strategy	To set gates, an untransfected HEK-293T control population was used. Gate 1 was set in the FSC/SSC plot to gate for the total starting cell population. Gate 2 was set in the BFP/SSC plot to gate for cells transfected with the indicated splicing reporters based on BFP fluorescence. Supplemental Figure 10 visually exemplifies the gating strategy.

☒ Tick this box to confirm that a figure exemplifying the gating strategy is provided in the Supplementary Information.
